# Adolescent cognitive function and risk of gestational diabetes mellitus: A retrospective population-based cohort study

**DOI:** 10.1371/journal.pone.0351780

**Published:** 2026-07-17

**Authors:** Tomer Talmy, Tali Cukierman-Yaffe, Shali Mazaki-Tovi, Estela Derazne, Dorit Tzur, Arnon Afek, Avi Shina, Gabriel Chodick, Gilad Twig, Rakefet Yoeli-Ullman

**Affiliations:** 1 Israel Defense Forces Medical Corps, Surgeon General’s Headquarters, Ramat Gan, Israel; 2 Department of Military Medicine and “Tzameret”, Faculty of Medicine, Hebrew University of Jerusalem, Jerusalem, Israel; 3 Division of Anesthesia, Intensive Care & Pain Management, Tel Aviv Sourasky University Medical Center, Gray Faculty of Medicine and Health Sciences, Tel Aviv University, Tel Aviv, Israel; 4 Gertner Institute for Epidemiology and Health Policy Research, Sheba Medical Center Tel Hashomer, Ramat Gan, Israel; 5 Division of Endocrinology, Diabetes and Metabolism, Sheba Medical Center, Tel Hashomer, Ramat Gan, Israel; 6 Gray Faculty of Medicine and Health Sciences, Tel Aviv University, Tel Aviv, Israel; 7 Department of Epidemiology and Preventive Medicine, School of Public Health, Gray Faculty of Medical and Health Sciences, Tel Aviv University, Tel Aviv, Israel; 8 Department of Obstetrics and Gynecology, Sheba Medical Center, Tel Hashomer, Ramat Gan, Israel; 9 The Dina Recanati School of Medicine, Reichman University, Herzliya, Israel; 10 Maccabitech Institute for Research and Innovation, Maccabi Healthcare Services, Tel Aviv, Israel; 11 Incumbent of the Hella Gertner Chair for Research in Hypertension, Gray Faculty of Medicine and Health Sciences, Tel Aviv University, Tel Aviv, Israel; University of Melbourne, AUSTRALIA

## Abstract

**Background:**

Gestational diabetes mellitus (GDM) is increasingly prevalent and linked with adverse maternal and neonatal outcomes. Low cognitive function in youth has been associated with various adverse metabolic outcomes. This study examined the association between adolescent cognitive function and GDM in the first pregnancy.

**Methods:**

In this retrospective nationwide population-based cohort study, data from the Israel Defense Forces conscription database, including cognitive assessments conducted at approximately age 17 years (1976–2016), were linked with electronic medical records from Maccabi Healthcare Services, documenting prenatal care and gestational diabetes screening data. General intelligence test (GIT) scores were standardized into sex-specific Z-scores and categorized as low, intermediate, or high. GDM during first pregnancy was defined according to the two-step approach. Logistic regression analyses were used to calculate odds ratios (OR) for GDM.

**Results:**

Among 189,663 women, 21,979 (11.6%) had low, 130,215 (68.7%) had intermediate, and 37,469 (19.8%) high GIT Z-scores; 10,187 (5.4%) developed GDM. Relative to high scores, low and intermediate scores were associated with higher odds of GDM: OR 1.17 (95% CI 1.08–1.26) and OR 1.09 (1.03–1.15), respectively.

**Conclusions:**

Lower adolescent cognitive function was modestly associated with increased risk of GDM in the first pregnancy, independent of sociodemographic factors and adolescent BMI. Cognitive function may serve as an early marker of maternal metabolic health.

## Introduction

Gestational diabetes mellitus (GDM) is a significant health concern, affecting approximately 1 in 7 pregnancies globally, and is linked to numerous adverse maternal and neonatal outcomes, including macrosomia, birth trauma, and an increased risk of operative delivery [1–3]. GDM has also been associated with negative long-term consequences for both the mother and offspring. Individuals diagnosed with GDM have an elevated risk of subsequently developing type 2 diabetes mellitus and cardiovascular disease [1,4,5]. Offspring of individuals diagnosed with GDM during pregnancy are also at increased risk of developing cardiometabolic disorders later in life [1,2,6]. Although the global prevalence of GDM ranges widely due to differing diagnostic criteria and screening policies, it has emerged as a growing public health issue [2,6].

Differences in cognitive function have been linked with a range of adverse health outcomes, including all-cause mortality, metabolic impairment (i.e., type 2 diabetes mellitus) and cardiovascular disease [7–10]. Several mechanisms may underlie this association, including health-related behaviors, adherence to preventive care, socioeconomic trajectories, and shared developmental or biological determinants that may influence both cognitive and metabolic health [11,12].

Since GDM may represent an early clinical manifestation of underlying insulin resistance and metabolic vulnerability, [13] it is plausible that adolescent cognitive function may also be associated with the risk of GDM. However, this relationship has yet to be investigated. Examining the association between adolescent cognitive performance and later GDM may thereby provide further insight into the increasingly recognized interplay between cognition and metabolic health. Therefore, in this study, we aimed to evaluate the association between late adolescent cognitive function and subsequent GDM during the first pregnancy among a large population-based cohort.

## Materials and methods

### Study design and population

This is a population-based retrospective cohort study linking data from Israel Defense Forces (IDF) conscription database between 1976 and 2016 and records of prenatal care from a single health maintenance organization (Accessed December 4, 2023). Military service is mandatory in Israel, and adolescents undergo a battery of tests at the age of 17 years to determine their qualifications for military service. Data collected during these evaluations, which includes demographic information, medical evaluation and cognitive assessments, has been systematically documented since 1967. After completing military service, all individuals are insured and receive their healthcare services from one of the four state-mandated civilian health maintenance organizations. Maccabi Health Services (MHS) is Israel’s second largest health maintenance organization, insuring approximately a quarter of the Israeli population [14]. The study included all women in the MHS database who had undergone adolescent prerecruitment evaluation and later had a documented pregnancy with gestational diabetes screening using the glucose challenge test (GCT), as previously described [15]. Exclusion criteria included missing records of adolescent body mass index or cognitive function score, diagnosis of diabetes documented prior to the IDF’s prerecruitment assessment, diagnosis of pregestational diabetes before first documented pregnancy in MHS, or incomplete GCT due to technical issues.

The primary outcome of this study was diagnosis of GDM during the first pregnancy documented in the MHS database. This study adhered to the Strengthening the Reporting of Observational Studies in Epidemiology (STROBE) reporting guideline [16]. The study protocol was approved by the Institutional Review Boards of the Israel Defense Forces Medical Corps (2018−1860) and Maccabi Health Services (0122-19-MHS).

### Baseline demographics

Data on individuals’ demographics, adolescent BMI, blood pressure and cognitive function scores were obtained from documentation of the compulsory pre-recruitment assessment. This assessment includes a medical evaluation and physical examination by a physician, review of previous medical history, as well as assessment and measurements of weight, height and blood pressure, recorded by trained medics during the same day [17,18]. Adolescent body mass index was categorized into four subgroups, as previously performed and validated on Israeli adolescents: Underweight (BMI < 5^th^ percentile), normal (5^th^ ≤ BMI < 85^th^ percentiles), overweight (85^th^ ≤ BMI < 95^th^ percentiles), obese (≥95^th^ percentile) [15,19,20]. Demographic data collected included birth year, education, and residential socioeconomic status. Year of birth was categorized by decades (1960–69, 1970–79, 1980–89, 1990–2000). Education was dichotomized into two categories ≤11 years or 12 years of formal schooling, with the latter corresponding to completion of high school, as previously reported [10,15]. Residential socioeconomic status ranges on an ordinal scale of 1–10, reflecting the socioeconomic characteristics of each locality in Israel, as defined by the Israeli Bureau of Statistics. Socioeconomic status was classified into three categories: low (1–4), intermediate (5–7) and high (8–10), as detailed in previous investigations [21]. Maternal age at first pregnancy was categorized into five groups based on 5-year intervals (18–24, 25–29, 30–34, 35–39, 40 and above).

### Cognitive assessment

The general intelligence test (GIT) is administered as part of the prerecruitment assessment and overseen by qualified and trained personnel. The four subdomains of the multiple-choice assessment include: Raven’s Progressive Matrices-R, measuring nonverbal abstract reasoning and visual-spatial problem-solving abilities; Similarities-R, measuring verbal abstraction skills and categorization; Otis-R, measuring verbal intelligence by assessing the ability to understand and carry out verbal instructions; and Arithmetic-R, measuring mathematical reasoning skills, concentration, and concept manipulation [8,10]. The summed scores of the four sub-tests included in the assessment is totaled into a final score on a 90-point scale, which has been validated as a global measure of overall intelligence [22]. The final score has also demonstrated high correlation (r > 0.8) with the total intelligence quotient as assessed by the Wechsler Adult Intelligence Scale [8,10,23–26]. This measure has been widely used in several studies investigating the relationship between cognitive function and various healthcare outcomes [8–10,27–29].

To improve comparability to previous studies [7] and normalize cognitive assessment scores throughout the study period, we converted subjects’ GIT scores to sex-specific Z-scores reflecting relative performance versus other females for each calendar year [8]. These were categorized into three categories: (1) low – GIT Z-score < −1; (2) intermediate – GIT Z-score between −1 and 1; (3) high – GIT Z-score> 1, serving as the primary independent variable in this study.

### Gestational diabetes mellitus screening data

The study accessed data on prenatal follow-up from MHS which has maintained a digital database recording the results of prenatal screening for GDM since 2001, primarily employing the two-step screening approach [30]. This approach consists of a 50-g-GCT and 100-g-oral glucose tolerance test (OGTT), indicated for women with abnormal GCT results (≥ 140 mg/dL; 7.8 mmol/L). Both tests are included in Israel’s national screening program, and covered free of charge by National Health Insurance plans [30].

GDM was diagnosed according to the criteria set by the National Diabetes Data Group [31] by either a 50 gr-GCT ≥ 200 mg/dL (11.1 mmol/L) or at least two abnormal values in the OGTT performed for women with GCT scores ≥140 mg/dL (7.8 mmol/L) but less than 200 mg/dL (11.1 mmol/L). Cutoff values for abnormal OGTT were consistent with the Carpenter and Coustan thresholds [31]: fasting serum glucose concentration ≥ 95 mg/dL (5.3 mmol/L), one hour serum glucose concentration ≥ 180 mg/dL (10.0 mmol/L), two hours serum glucose concentration ≥ 155 mg/dL (8.6 mmol/L), and three-hours serum glucose concentration ≥ 140 mg/dL (7.8 mmol/L).

### STATISTICAL ANALYSIS

Continuous variables are described as means ± standard deviations and categorical variables summarized as numbers and percentages. Differences in variables between the GIT Z-score groups were compared using chi-square and analysis of variance or Kruskal-Wallis tests as appropriate. Logistic regression analyses were used to calculate odds ratios (OR) and 95% confidence intervals (CI) for the incidence of GDM, using high GIT Z-score as reference. First, an unadjusted analysis of the association between GIT Z-score and the outcome of GDM was performed (Model 1). This analysis was then repeated, adjusting for maternal age at pregnancy, regarded as one of the most important risk factors for GDM (Model 2) [1,2,15,32]. Thereafter, multivariable logistic regression was performed, including the following prespecified covariates: (1) maternal birth year, (2) education status, (3) residential socioeconomic status category, (4) maternal adolescent BMI category and (5) maternal age at first pregnancy (Model 3). Missing data were not imputed, and therefore, observations with missing data for any variable included in a given model were excluded from the aforementioned analysis. Analyses were performed using IBM-SPSS (version 25.0) and R software version 4.2.1 (R Foundation for Statistical Computing, Vienna, Austria).

### Subgroup And Sensitivity Analyses

Several sub-analyses were performed. First, the main analysis was repeated for individuals with unimpaired health at adolescence (i.e., no documentation of chronic comorbidities, malignancy or major operation indicating fitness for combat service), as previously conducted [8,9]. Second, the main analysis was repeated for individuals with continuous insurance coverage in MHS immediately following discharge from military service, to account for potential misclassification of first pregnancy which may have been covered by a different health maintenance organization before transferring to MHS, thus absent from the study database. Next, a subgroup analysis was conducted for individuals with available pre-pregnancy BMI data, defined as any BMI measurement recorded in the MHS database within three years prior to pregnancy. Baseline characteristics of this subgroup were compared with those of individuals without available pre-pregnancy BMI data. Logistic regression analyses were then repeated within this group, first adjusting for pre-pregnancy BMI and maternal age at pregnancy, followed by a multivariable model including the covariates used as described for the primary analysis. In addition, the adjusted analysis was repeated after stratifying the cohort by adolescent BMI category. Finally, a linear regression model was fitted to assess the linear relationship between GIT Z-score and GDM incidence, with GIT Z-score treated as a continuous variable.

## Results

### Baseline characteristics of the study population

**Fig 1:**
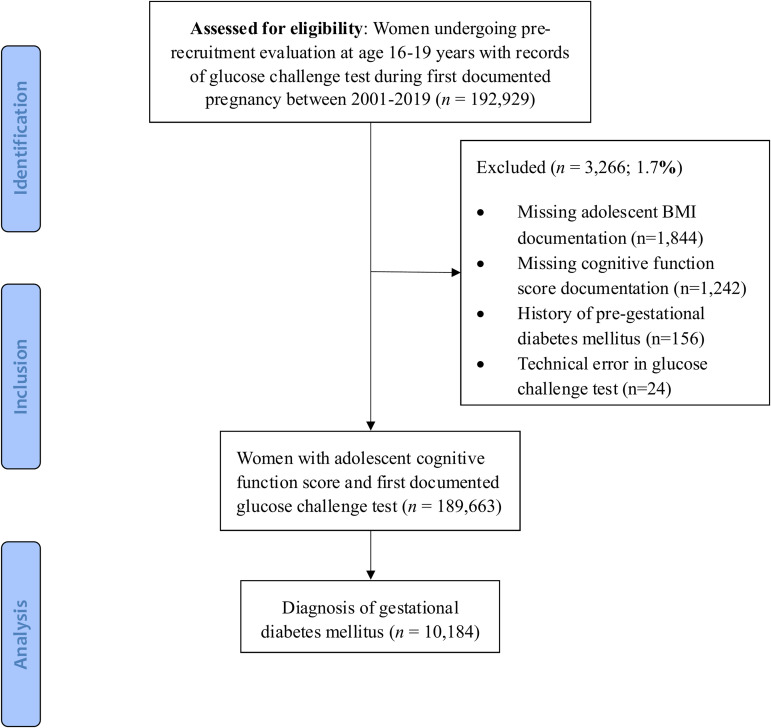
Flow chart of the study population. BMI- body mass index.

Of the 192,929 individuals who were assessed for eligibility 3,266 (1.7%) were excluded from the study (Fig 1). The final study population consisted of 189,663 individuals. Of these, 21,979 (11.6%), 130,215 (68.7%) and 37,469 (19.8%) belonged to the low, intermediate and high GIT Z-score groups, respectively.

Table 1 depicts the baseline characteristics of the study population, grouped by GIT Z-score. Mean age at first assessment was 17.3 ± 0.4 years. The proportion of women with obese adolescent BMI was 3.7% in the low GIT Z-score group, 2.2% in the intermediate group and 1.5% in the high group (p < 0.001). Participants with higher GIT Z-scores had a lower proportion of obesity in adolescence and higher proportions of ≥12 years of formal education, high residential socioeconomic status, and unimpaired health. Specifically, the proportions of obese adolescent BMI were 3.7%, 2.2%, and 1.5% across the low, intermediate, and high GIT groups, respectively (p < 0.001); the corresponding PROPORTIONS for ≥12 years of formal education were 91.9%, 98.2%, and 99.5% (p < 0.001), for high residential Socioeconomic status 19.6%, 29.7%, and 38.0% (p < 0.001), and for unimpaired health 69.7%, 74.1%, and 75.2% (p < 0.001).

**Table 1:** Baseline characteristics of the study population, grouped by general intelligence test (GIT) Z-score groups.

	General Intelligence Test Z-score				
	Low(Z-Score < −1)n = 21,979 (11.6%)	Intermediate(−1 < Z-Score < 1)n = 130,215 (68.6%)	High(Z-score > 1)n = 37,469 (19.8%)	Totaln = 189,663	p-value
***Year of birth***					
1960−69	2483 (11.3)	16854 (12.9)	4702 (12.5)	24039 (12.7)	<0.001
1970−79	7391 (33.6)	59343 (45.6)	17346 (46.3)	84080 (44.3)	
1980−89	8288 (37.7)	44936 (34.5)	13234 (35.3)	66458 (35.0)	
1990-2000	3817 (17.4)	9082 (7.0)	2187 (5.8)	45086 (8.0)	
**Age at first assessment**, years	17.4 ± 0.5	17.3 ± 0.4	17.3 ± 0.4	17.3 ± 0.4	<0.001
***Years of education***					
<12	1779 (8.1)	2393 (1.8)	179 (0.5)	4351 (2.3)	<0.001
≥12	20198 (91.9)	127804 (98.2)	37284 (99.5)	185286 (97.7)	
***Socioeconomic status***					
Low	4756 (21.7)	20228 (15.6)	4853 (13.1)	29837 (15.8)	<0.001
Medium	12861 (58.7)	70857 (54.7)	18206 (49.0)	101924 (54.0)	
High	4292 (19.6)	38436 (29.7)	14120 (38.0)	56848 (30.1)	
**Height** (cm)	161.3 ± 6.2	162.5 ± 6.1	163.4 ± 6.0	162.6 ± 6.1	<0.001
**Systolic BP** (mmHg)	111.9 ± 11.9	112.1 ± 11.7	112.2 ± 11.8	112.1 ± 11.8	0.016
**Diastolic BP** (mmHg)	70.5 ± 8.2	70.7 ± 8.1	70.7 ± 8.0	70.7 ± 8.1	<0.001
**Unimpaired health**	15311 (69.7)	96428 (74.1)	28181 (75.2)	139920 (73.8)	<0.001
**Israeli born**	18652 (84.9)	108721 (83.5)	29451 (78.6)	156824 (82.7)	<0.001
***Adolescent BMI (kg/m*** ^***2***^ **)**					
Mean BMI	21.8 ± 3.7	21.4 ± 3.2	21.3 ± 2.9	21.4 ± 3.2	<0.001
Underweight	1245 (5.7)	6111 (4.7)	1522 (4.1)	8878 (4.7)	<0.001
Normal weight	17640 (80.3)	110439 (84.8)	32714 (87.3)	160793 (84.8)	
Overweight	2289 (10.4)	10842 (8.3)	2656 (7.1)	15787 (8.3)	
Obese	805 (3.7)	2823 (2.2)	577 (1.5)	4205 (2.2)	
***Age at first pregnancy, years***					
Mean age	29.4 ± 5.1	31.1 ± 4.7	31.6 ± 4.3	31.0 ± 4.7	<0.001
18–24	4334 (19.7)	9746 (7.5)	1382 (3.7)	15462 (8.2)	<0.001
25–29	8702 (39.6)	49615 (38.1)	13340 (35.6)	71657 (37.8)	
30–34	5708 (26.0)	45129 (34.7)	15130 (40.4)	65967 (34.8)	
35–39	2588 (11.8)	20007 (15.4)	5922 (15.8)	28517 (15.0)	
40–49	647 (2.8)	5718 (4.4)	1695 (4.5)	8060 (4.2)	
**Gestational age at first GDM screening**, weeks*	25.0 (24.1-26.1)	24.9 (24.0-26.0)	24.9 (24.0-25.9)	24.9 (24.0-26.0)	<0.001

Dichotomous variables are reported as N (%), continuous variables are reported as mean ± standard deviation or median (IQR), as appropriate.

GDM, gestational diabetes mellitus, BMI, body mass index; BP, blood pressure; BMI categories: underweight (BMI < 5th percentile), normal (5th ≤ BMI < 85th percentiles), overweight (85th ≤ BMI < 95th percentiles), obese (≥95th percentile).

*Data regarding the timing of the first GDM examination were missing for 4,389 (20.0%) participants in the Low Z-score group, 23,026 (17.7%) in the Intermediate group, and 7,142 (19.1%) in the High Z-score group.

### Association between adolescent cognitive function and GDM

Overall, 10,187 (5.4%) individuals were diagnosed with GDM, with a rate of 5.5% (n = 1,209), 5.4% (n = 7,079) and 5.0% (n = 1,899) for women in the low, intermediate and high GIT Z-score groups, respectively (p-trend<0.001).

**Fig 2:**
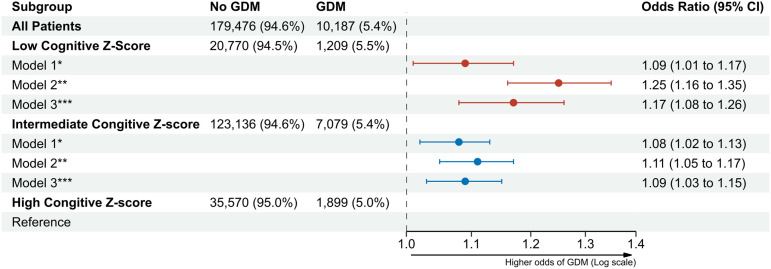
Logistic regression models for the relationship between general intelligence test (GIT) Z-score groups and incidence of gestational diabetes mellitus (GDM). Reference Category for GIT Z-score is high (>1). GDM – gestational diabetes mellitus; BMI- body mass index. *Model 1- unadjusted. ** Model 2- adjusted for maternal age at pregnancy. ***Model 3- adjusted for maternal year of birth, education category, residential socioeconomic status category, adolescent BMI category and maternal age at pregnancy. Overall, 1,080 observations (0.6%) had missing data for at least one covariate and were therefore excluded from the final model.

Fig 2 presents the results of the logistic regression analysis examining the association between GIT Z-score and incidence of GDM. The unadjusted analysis demonstrated significant ORs for the development of GDM among women with low 1.09 (95% CI 1.01–1.17) and intermediate 1.08 (95% CI 1.02–1.13), compared with high GIT Z-scores (Model 1). These associations remained statistically significant after adjustment for maternal age at pregnancy (Model 2 – Low: 1.25, 95% CI 1.16–1.35; Intermediate: 1.11, 95% CI 1.05–1.17) and after adjustment for maternal year of birth, education category, residential socioeconomic status category, adolescent BMI category and maternal age at pregnancy (Low: 1.17, 95% CI 1.08–1.26; Intermediate: 1.09, 95% CI 1.03–1.15; Model 3).

These associations persisted in the sensitivity analyses conducted for women with unimpaired health (S1 Fig) and those with continuous insurance in MHS during the follow-up period (S2 Fig). Pre-pregnancy BMI data were available for 58,456 (30.8%) of the individuals included in the study, more often for individuals from recent decades (S1 Table). When repeating the regression analysis in this sub-population, with the addition of pre-pregnancy BMI category to the regression models the association between low GIT Z-score and incidence of GDM lost its statistical significance. The association remained statistically significant for the intermediate GIT Z-score (S3 Fig). In analyses stratified by adolescent BMI groups (S4 Fig), estimates of the association between lower GIT Z-score and GDM were most pronounced among women with overweight and obesity, whereas weaker and non-significant associations were observed in the underweight group.

## Discussion

This population-based cohort study demonstrates an inverse association between cognitive function assessed at late adolescence and subsequent GDM during the first pregnancy. The association remained significant after adjustment for potential confounders including maternal year of birth, socioeconomic status, maternal education, adolescent BMI and maternal age at pregnancy (Low: 1.17, 95% CI 1.08–1.26; Intermediate: 1.09, 95% CI 1.03–1.15; as compared with high GIT Z-score). The association also persisted among individuals with unimpaired health at adolescence.

The findings of the current study build upon prior research that has examined the relationship between adolescent cognitive function and metabolic outcomes, including type 2 diabetes mellitus [10,33]. Our results are consistent with and expand on previous findings from Danish [34], British [33,35] and Vietnamese [36] cohorts which reported that lower cognitive function in adolescence was associated with an increased risk of various dysglycemic outcomes such as type 2 diabetes, elevated glycated hemoglobin, or higher blood glucose levels. Reported point estimates for these outcomes in these studies ranged from approximately 1.4 to 1.7 for individuals with lower cognitive function as compared with normal or high function. These findings have also been demonstrated in several cohorts of Israeli individuals, relying on the same prerecruitment cognitive assessment used in this study. In a nationwide study of 971,677 Israeli adolescents, lower cognitive function scores were associated with an approximately two-fold increase in risk of early-onset type 2 diabetes in both men and women, despite adjustment for socioeconomic status [10]. In a study of the Metabolic Lifestyle and Nutrition Assessment in Young Adults cohort of permanent service Israeli military personnel, lower cognitive function in late adolescence was independently associated with an increased risk of impaired fasting glucose in young adulthood (Hazard ratio 1.8; 95% CI, 1.4–2.3) [37].

Although type 2 diabetes mellitus and GDM share several underlying pathophysiological mechanisms, [2,38] GDM remains a distinct entity which may often present as the first clinical manifestation of dysglycemia. To the best of our knowledge this is the first study evaluating the association between late adolescent cognitive function and the risk for subsequent GDM. While the associations observed in our study were weaker than those reported in studies examining type 2 diabetes mellitus, this may be explained by the more heterogeneous and transient nature of GDM, which is shaped not only by underlying metabolic vulnerability but also by pregnancy-specific physiological changes and obstetric factors [39,40]. In addition, GDM occurs earlier in the natural history of dysglycemia and may therefore reflect a less pronounced metabolic phenotype than overt type 2 diabetes. Nevertheless, the consistency of our findings across multiple adjusted models and sensitivity analyses suggests that, at the population level, lower cognitive function may indeed be associated with risk of GDM. Given the growing body of evidence linking glucose intolerance in pregnancy with future maternal [1,4,5] and child [41–43] cardiometabolic health, the findings of the current study provide additional support to the concept that cognitive function during adolescence could serve as an early indicator for metabolic risk.

The observed association between late adolescent cognitive function and incidence of GDM could have several explanations. First, this association may be principally mediated by the link between cognitive function and social determinants of health including socioeconomic status and maternal education. Low socioeconomic status has been previously linked with adverse pregnancy outcomes, including but not limited to GDM [44,45]. Although our results remained consistent when controlling for various covariates, including socioeconomic status and education, residual confounding by unmeasured socioeconomic factors may also play a role in mediating this relationship. Moreover, because these variables were assessed in adolescence, they may not fully capture socioeconomic circumstances later in adulthood or at the time of pregnancy.

Health literacy and adoption of health promoting behaviors may also facilitate the relation between late adolescent cognitive function and incidence of GDM. Previous studies have shown that cognitive function in childhood may impact dietary behaviors and engagement in physical exercise, [7,46] which could potentially influence the risk of GDM [47]. Weight gain in particular, both before and during gestation has been shown to augment the risk of GDM [15,48]. Disparities in health literacy may contribute to suboptimal adherence to prenatal lifestyle recommendations aimed at reducing the risk of GDM, or to reduced attendance at first-trimester prenatal visits where such recommendations are commonly provided. The described association may also be explained by biochemical pathways or mutually inherited traits affecting both cognitive function and the underlying pathophysiology of GDM. Factors such as leptin, which is secreted by the placenta during pregnancy with increased production in GDM, [38] and is thought to play a role in cognition [49], may be involved in the underlying mechanism. Additionally, GDM has been associated with impaired insulin signaling, altered placental gene expression, vascular changes and variations in neurohormonal networks, [38,50] all of which may suggest a multifactorial pathophysiology that may also be involved in the brain and cognitive development. Finally, cognitive decline in the perinatal and post-natal periods has also been linked to GDM, potentially demonstrating a bi-directional relationship which is mediated by shared mechanisms [50,51].

The findings of this study have potential public health implications, particularly in the context of the rising global rates of GDM [6,52] and the increasingly appreciated long-term health implications of GDM on mothers and their offspring [1,2]. Recognizing cognitive function at adolescence as a potential risk factor for GDM may allow for targeted interventions aiming to improve health literacy and promote lifestyle changes. Moreover, these findings shed further light on the link between adolescent global cognitive function and a range of adverse health outcomes, [7,10,33] suggesting that early investments in education and efforts to reduce disparities may promote positive long-term health benefits in adulthood.

This study has several limitations. First, data on pre-pregnancy BMI were available for only 30.8% of the cohort. In the sensitivity analysis including individuals with available pre-pregnancy BMI the association between intermediate GIT Z-score and incidence of GDM remained statistically significant, even after adjusting for possible confounders, including pre pregnancy BMI. However, the association between low GIT Z-score and incidence of GDM lost its significance. This may be due to the small study sample available for this analysis. Second, the diagnosis of GDM in this study relied on the results of the two-step screening strategy. Although it was previously reported that this strategy was used for over 90% of women insured by MHS, [30] women directly referred to a one-step 75-g or 100-g OGTT were not included in our cohort. Third, despite mandatory military service in Israel, certain ethnic groups—including Druze, Arab, and ultra-Orthodox Jewish individuals—are generally exempt, leading to underrepresentation of these populations. Fourth, education level was assessed at the completion of high school, and we had no data on higher education attainment. Fifth, although an association was observed between overall cognitive score and GDM, data on specific cognitive subdomains were unavailable, precluding analysis of individual GIT components, which may have distinct relationships with metabolic risk. Sixth, several potentially relevant determinants of GDM risk, including family history of cardiometabolic disease, were unavailable for adjustment. In addition, although we adjusted for multiple socioeconomic covariates, these were measured in adolescence and therefore may not fully capture socioeconomic conditions later in adulthood or at the time of pregnancy. Likewise, cognitive function was assessed once during adolescence, several years before pregnancy, and this single measurement may not fully reflect cognitive function at the time of pregnancy.

This study’s strengths include the use of two large nationwide databases: the IDF compulsory prerecruitment assessment database including systematic adolescent sociodemographic data, medical evaluation and cognitive function assessed using a general intelligence test; and the MHS digital database recording the results of prenatal screening for GDM which has been shown to have high compliance rates in this cohort [30]. Notably, prior analyses using our dataset have demonstrated associations between cognitive function and various metabolic outcomes, with point estimates comparable to those reported in other European cohorts, thus strengthening the external validity of our findings in the context of other Western populations [7].

## Conclusions

In this population-based cohort study, lower cognitive function in late adolescence was associated a modestly increased risk of developing GDM during the first pregnancy. This association remained robust after adjustment for a range of sociodemographic and clinical covariates. These findings suggest that cognitive function may serve as an early marker of maternal metabolic risk which can inform targeted interventions aimed at improving health literacy and promoting health-seeking behaviors among women of reproductive age.

### Glossary

**GIT (General Intelligence Test)** – A standardized cognitive assessment administered during Israel’s military pre-recruitment process, comprising four subtests (Raven’s, Otis-R, Similarities-R, and Arithmetic-R). The summed score serves as a measure of global intelligence.
**Israel Defense Forces (IDF)** – The military forces of the State of Israel. Mandatory service includes a standardized prerecruitment assessment used as a data source in this study.
**MHS (Maccabi Healthcare Services)** – One of the four state-mandated health maintenance organizations in Israel, providing healthcare services to approximately 25% of the population.
**Socioeconomic Status (SES)** – An index that reflects the economic and social position of individuals by localities. Israeli localities are scored on a scale of 1–10.
**Z-score** – Statistical measure that describes a value’s position relative to the mean of a group, expressed in terms of standard deviations. In this study, Z-scores were utilized to standardize cognitive scores by sex and year.

## Supporting information

S1 Fig Sensitivity analysis limited to women with unimpaired health at adolescence. Logistic regression models for the relationship between general intelligence test (GIT) Z-score groups and incidence of gestational diabetes mellitus (GDM) limited to individuals with unimpaired health at adolescence. Unimpaired health at adolescence is defined as no documentation of chronic comorbidities, malignancy or major operation indicating fitness for combat service Reference category for GIT Z-score is high (>1). GDM – gestational diabetes mellitus; BMI- body mass index. *Model 1- unadjusted. ** Model 2- adjusted for maternal age at pregnancy. ***Model 3- adjusted for maternal year of birth, education category, residential socioeconomic status category, adolescent BMI category and maternal age at pregnancy.
(PDF)

S2 Fig Sensitivity analysis limited to women with continuous membership in Maccabi Health Services. Logistic regression models for the relationship between general intelligence test (GIT) Z-score groups and incidence of gestational diabetes mellitus (GDM) limited to individuals with continuous membership in Maccabi Health Services immediately following Discharge from military service. Reference category for GIT Z-score is high (>1). GDM – gestational diabetes mellitus; BMI- body mass index. *Model 1- unadjusted. ** Model 2- adjusted for maternal age at pregnancy. ***Model 3- adjusted for maternal year of birth, education category, residential socioeconomic status category, adolescent BMI category and maternal age at pregnancy.
(PDF)

S3 Fig Sensitivity analysis of women with pre-pregnancy BMI. **D**epicted are Logistic regression models for the relationship between general intelligence test (GIT) Z-score groups and incident gestational diabetes mellitus (GDM) limited to individuals for whom pre-pregnancy BMI was available. Pre-pregnancy BMI was defined as BMI recorded in the Maccabi Health Services database in the three years preceding the first pregnancy. Reference category for GIT Z-score is high (>1). GDM – gestational diabetes mellitus; BMI- body mass index. *Model 1- unadjusted. ** Model 2- adjusted for maternal age at pregnancy and pre pregnancy BMI category. ***Model 3- adjusted for maternal year of birth, education category, residential socioeconomic status category, adolescent BMI category, pre pregnancy BMI category and maternal age at pregnancy.
(PDF)

S4 Fig Stratified analysis of the association between late adolescent cognitive function and gestational diabetes mellitus (GDM) by adolescent BMI category. BMI categories were defined as underweight (BMI < 5th percentile), normal weight (5th ≤ BMI < 85th percentile), overweight (85th ≤ BMI < 95th percentile), and obese (BMI ≥ 95th percentile). Within each BMI stratum, adjusted logistic regression models estimated the association between general intelligence test (GIT) Z-score group and incident GDM, using the high GIT Z-score group as the reference category. Models were adjusted for maternal year of birth, education category, residential socioeconomic status category, and maternal age at pregnancy.
(PDF)

S1 Table Comparison of demographics and baseline characteristics of women in the study population for whom pre-pregnancy BMI data were available vs. those without pre-pregnancy BMI data. Dichotomous variables are reported as N (%), continuous variables are reported as mean ± standard deviation. GDM, gestational diabetes mellitus, BMI, body mass index; BP, blood pressure; BMI categories: underweight (BMI < 5th percentile), normal (5th ≤ BMI < 85th percentiles), overweight (85th ≤ BMI < 95th percentiles), obese (≥95th percentile); Abbreviations: BMI, body mass index; BP, blood pressure; GDM, gestational diabetes mellitus; GIT, General Intelligence Test; SD, standard deviation; cm, centimeters; mmHg, millimeters of mercury; N/A, not applicable.
(PDF)
