# Supplementary material for: Adolescent cognitive function and risk of gestational diabetes mellitus: A retrospective population-based cohort study
Source: PLoS One. 2026 Jul 17;21(7):e0351780. doi: 10.1371/journal.pone.0351780 (PMC13379011; doi:10.1371/journal.pone.0351780)

S1 Fig. Logistic regression models for the relationship between general intelligence test (GIT) Z-score groups and incidence of gestational diabetes mellitus (GDM) limited to individuals with unimpaired health at adolescence. Unimpaired health at adolescence is defined as no documentation of chronic comorbidities, malignancy or major operation indicating fitness for combat service Reference category for GIT Z-score is high (>1).

GDM – gestational diabetes mellitus; BMI- body mass index.

*Model 1- unadjusted.

** Model 2- adjusted for maternal age at pregnancy.

***Model 3- adjusted for maternal year of birth, education category, residential socioeconomic status category, adolescent BMI category and maternal age at pregnancy

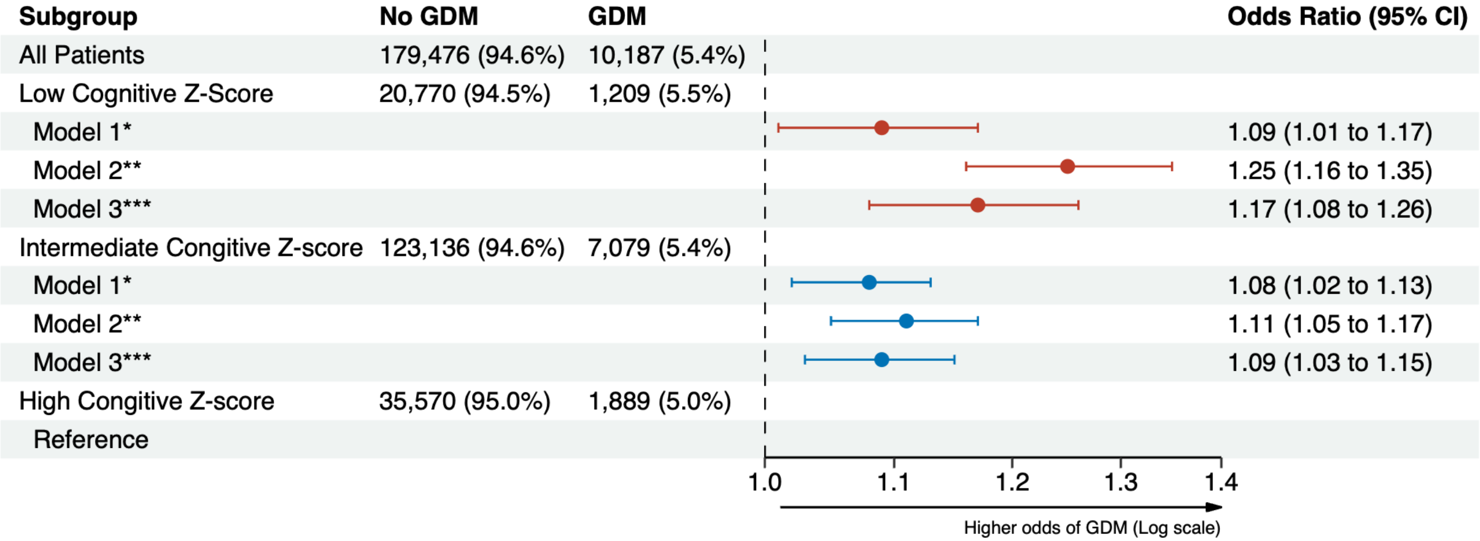

Supplement: S1 Fig — Logistic regression models for the relationship between general intelligence test (GIT) Z-score groups and incidence of gestational diabetes mellitus (GDM) limited to individuals with unimpaired health at adolescence. Unimpaired health at adolescence is defined as no documentation of chronic comorbidities, malignancy or major operation indicating fitness for combat service Reference category for GIT Z-score is high (>1). GDM – gestational diabetes mellitus; BMI- body mass index. *Model 1- unadjusted. ** Model 2- adjusted for maternal age at pregnancy. ***Model 3- adjusted for maternal year of birth, education category, residential socioeconomic status category, adolescent BMI category and maternal age at pregnancy. (PDF) [file pone.0351780.s001.pdf]
