# Supplementary material for: Adolescent cognitive function and risk of gestational diabetes mellitus: A retrospective population-based cohort study
Source: PLoS One. 2026 Jul 17;21(7):e0351780. doi: 10.1371/journal.pone.0351780 (PMC13379011; doi:10.1371/journal.pone.0351780)

S2 Fig. Logistic regression models for the relationship between general intelligence test (GIT) Z-score groups and incidence of gestational diabetes mellitus (GDM) limited to individuals with continuous membership in Maccabi Health Services immediately following Discharge from military service. Reference category for GIT Z-score is high (>1).

GDM – gestational diabetes mellitus; BMI- body mass index.

*Model 1- unadjusted.

** Model 2- adjusted for maternal age at pregnancy.

***Model 3- adjusted for maternal year of birth, education category, residential socioeconomic status category, adolescent BMI category and maternal age at pregnancy

Subgroup	No GDM	GDM	Odds Ratio (95% CI)
All Patients	164,073 (94.6%)	9,423 (5.4%)	
Low Cognitive Z-Score			
Model 1*	18,931 (94.4%)	1,120 (5.6%)	1.09 (1.03 to 1.15)
Model 2**			1.26 (1.17 to 1.37)
Model 3***			1.18 (1.09 to 1.28)
Intermediate Congitive Z-score	112,712 (94.5%)	6,564 (5.5%)	
Model 1*			1.10 (1.02 to 1.19)
Model 2**			1.12 (1.06 to 1.18)
Model 3***			1.10 (1.04 to 1.16)
High Congitive Z-score	32,430 (94.9%)	1,739 (5.1%)	
Reference			

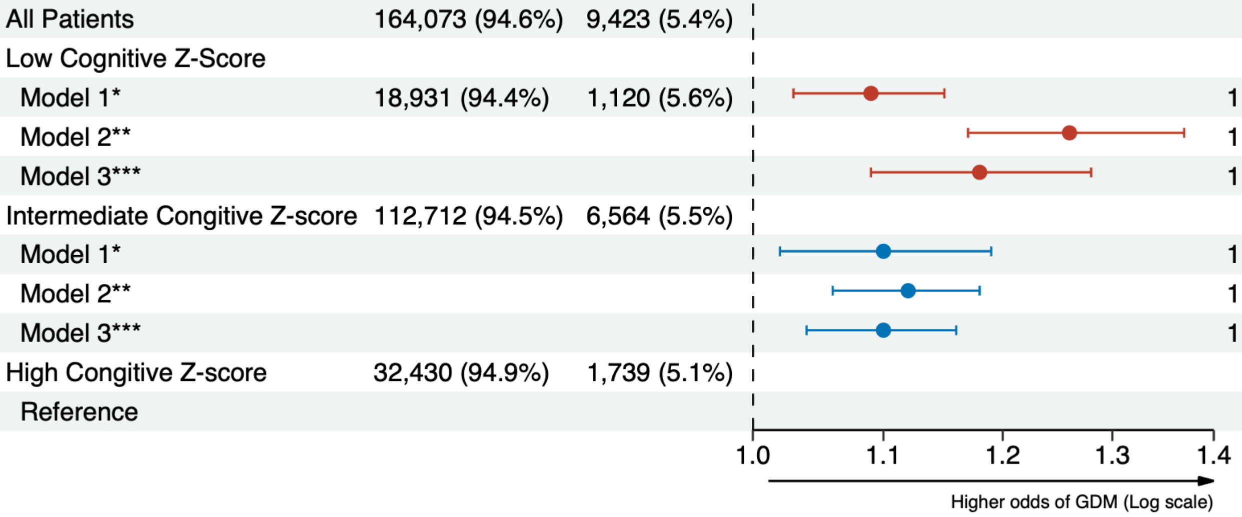

Supplement: S2 Fig — Logistic regression models for the relationship between general intelligence test (GIT) Z-score groups and incidence of gestational diabetes mellitus (GDM) limited to individuals with continuous membership in Maccabi Health Services immediately following Discharge from military service. Reference category for GIT Z-score is high (>1). GDM – gestational diabetes mellitus; BMI- body mass index. *Model 1- unadjusted. ** Model 2- adjusted for maternal age at pregnancy. ***Model 3- adjusted for maternal year of birth, education category, residential socioeconomic status category, adolescent BMI category and maternal age at pregnancy. (PDF) [file pone.0351780.s002.pdf]
