# Supplementary material for: Adolescent cognitive function and risk of gestational diabetes mellitus: A retrospective population-based cohort study
Source: PLoS One. 2026 Jul 17;21(7):e0351780. doi: 10.1371/journal.pone.0351780 (PMC13379011; doi:10.1371/journal.pone.0351780)

S3 Fig. Sensitivity analysis of women with pre-pregnancy BMI. Depicted are Logistic regression models for the relationship between general intelligence test (GIT) Z-score groups and incident gestational diabetes mellitus (GDM) limited to individuals for whom pre-pregnancy BMI was available. Pre-pregnancy BMI was defined as BMI recorded in the Maccabi Health Services database in the three years preceding the first pregnancy. Reference category for GIT Z-score is high (>1).

GDM – gestational diabetes mellitus; BMI- body mass index.

*Model 1- unadjusted.

** Model 2- adjusted for maternal age at pregnancy and pre pregnancy BMI category.

***Model 3- adjusted for maternal year of birth, education category, residential socioeconomic status category, adolescent BMI category, pre pregnancy BMI category and maternal age at pregnancy

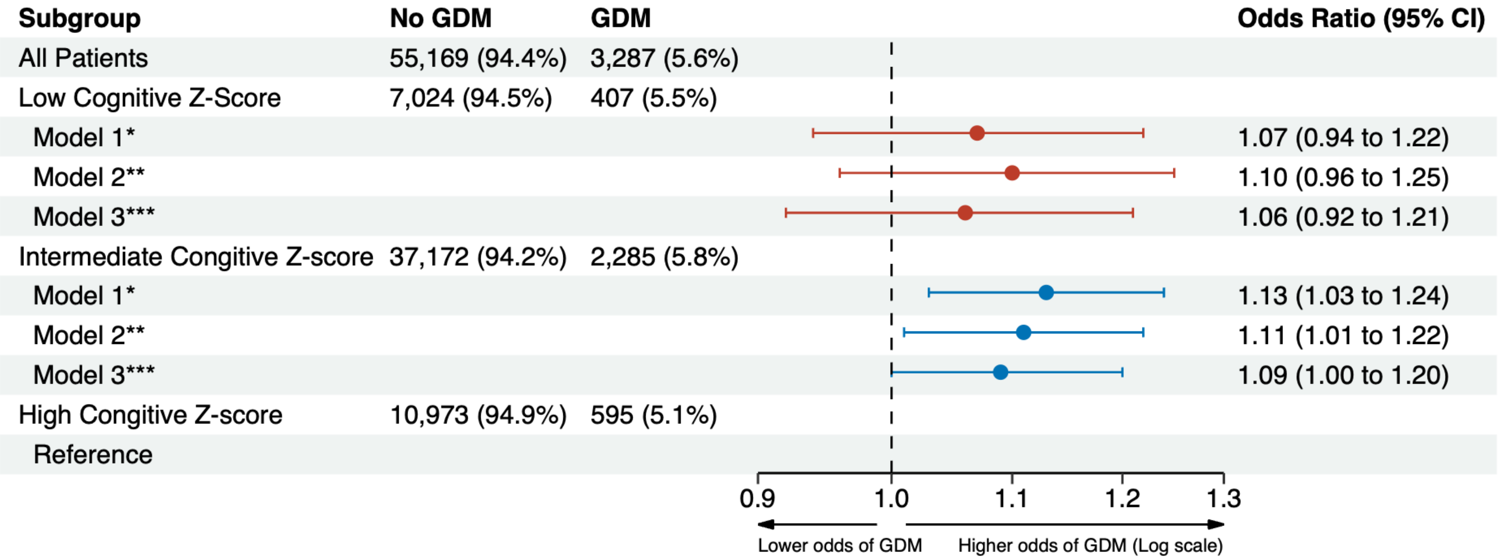

Supplement: S3 Fig — Depicted are Logistic regression models for the relationship between general intelligence test (GIT) Z-score groups and incident gestational diabetes mellitus (GDM) limited to individuals for whom pre-pregnancy BMI was available. Pre-pregnancy BMI was defined as BMI recorded in the Maccabi Health Services database in the three years preceding the first pregnancy. Reference category for GIT Z-score is high (>1). GDM – gestational diabetes mellitus; BMI- body mass index. *Model 1- unadjusted. ** Model 2- adjusted for maternal age at pregnancy and pre pregnancy BMI category. ***Model 3- adjusted for maternal year of birth, education category, residential socioeconomic status category, adolescent BMI category, pre pregnancy BMI category and maternal age at pregnancy. (PDF) [file pone.0351780.s003.pdf]
