# Supplementary material for: Adolescent cognitive function and risk of gestational diabetes mellitus: A retrospective population-based cohort study
Source: PLoS One. 2026 Jul 17;21(7):e0351780. doi: 10.1371/journal.pone.0351780 (PMC13379011; doi:10.1371/journal.pone.0351780)

S4 Fig. Stratified analysis of the association between late adolescent cognitive function and gestational diabetes mellitus (GDM) by adolescent BMI category. BMI categories were defined as underweight (BMI <5th percentile), normal weight (5th≤BMI<85th percentile), overweight (85th≤BMI<95th percentile), and obese (BMI ≥95th percentile). Within each BMI stratum, adjusted logistic regression models estimated the association between general intelligence test (GIT) Z-score group and incident GDM, using the high GIT Z-score group as the reference category. Models were adjusted for maternal year of birth, education category, residential socioeconomic status category, and maternal age at pregnancy.

Subgroup	No GDM	GDM	Odds Ratio (95% CI)
Underweight	8,337 (93.9%)	541 (6.1%)	
Low Cognitive Z-Score			1.16 (0.89 to 1.63)
Intermediate Congitive Z-score			1.24 (0.97 to 1.60)
Normal weight	152,669 (94.9%)	8,124 (5.1%)	
Low Cognitive Z-Score			1.12 (1.03 to 1.23)
Intermediate Congitive Z-score			1.07 (1.01 to 1.13)
Overweight	14,640 (92.7%)	1,147 (7.3%)	
Low Cognitive Z-Score			1.44 (1.15 to 1.79)
Intermediate Congitive Z-score			1.09 (0.92 to 1.29)
Obese	3,830 (91.1%)	375 (8.9%)	
Low Cognitive Z-Score			1.48 (0.96 to 2.29)
Intermediate Congitive Z-score			1.60 (1.13 to 2.32)

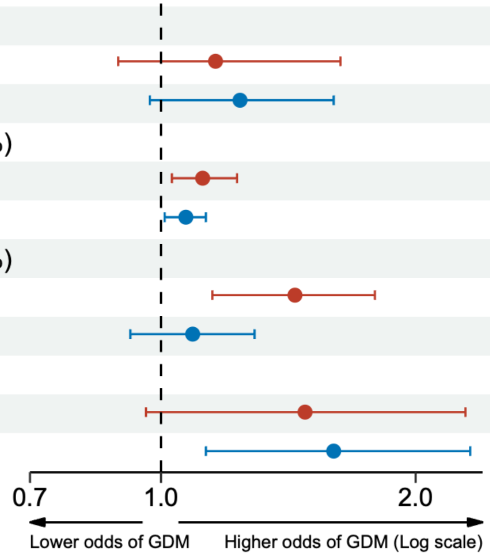

Supplement: S4 Fig — BMI categories were defined as underweight (BMI < 5th percentile), normal weight (5th ≤ BMI < 85th percentile), overweight (85th ≤ BMI < 95th percentile), and obese (BMI ≥ 95th percentile). Within each BMI stratum, adjusted logistic regression models estimated the association between general intelligence test (GIT) Z-score group and incident GDM, using the high GIT Z-score group as the reference category. Models were adjusted for maternal year of birth, education category, residential socioeconomic status category, and maternal age at pregnancy. (PDF) [file pone.0351780.s004.pdf]
