# Supplementary material for: Adolescent cognitive function and risk of gestational diabetes mellitus: A retrospective population-based cohort study
Source: PLoS One. 2026 Jul 17;21(7):e0351780. doi: 10.1371/journal.pone.0351780 (PMC13379011; doi:10.1371/journal.pone.0351780)
Supplement: S1 Table — Dichotomous variables are reported as N (%), continuous variables are reported as mean ± standard deviation. GDM, gestational diabetes mellitus, BMI, body mass index; BP, blood pressure; BMI categories: underweight (BMI < 5th percentile), normal (5th ≤ BMI < 85th percentiles), overweight (85th ≤ BMI < 95th percentiles), obese (≥95th percentile); Abbreviations: BMI, body mass index; BP, blood pressure; GDM, gestational diabetes mellitus; GIT, General Intelligence Test; SD, standard deviation; cm, centimeters; mmHg, millimeters of mercury; N/A, not applicable. (PDF) [file pone.0351780.s005.pdf]

S1 Table. Comparison of demographics and baseline characteristics of individuals in the study population for whom pre-pregnancy BMI data were available vs. those without pre-pregnancy BMI data.

	Availability of Pre-Pregnancy BMI			
	**Women without Pre-pregnancy BMI data** n (%) n = 131,207	**Women with Pre-pregnancy BMI data** n (%) n = 58,456	**Total** n (%) n = 189,663	**p-value**
Year of birth				
1960-69	22350 (17.0)	1689 (2.9)	24039 (12.7)	<0.001
1970-79	68869 (52.5)	15,211 (26.0)	84080 (44.3)	
1980-89	33256 (25.3)	33202 (56.8)	66458 (35.0)	
1990-2000	6732 (5.1)	8,354 (14.3)	15086 (8.0)	
Age at first assessment, year (M±SD)	17.3 ± 0.4	17.2 ± 0.4	17.3 ± 0.4	<0.001
Years of education				
<12	3186 (2.4)	1165 (2.0)	4351 (2.3)	<0.001
≥12	128001 (97.6)	57285 (98.0)	185286 (97.7)	
Socioeconomic status				
Low	20443 (15.7)	9404 (16.2)	29837 (15.8)	<0.001
Medium	69800 (53.5)	32124 (55.2)	101924 (54.0)	
High	40204 (30.8)	16644 (28.6)	56848 (30.1)	
**Height** (cm)	162.6 ± 6.1	162.5 ± 6.2	162.6 ± 6.1	0.008
**Systolic BP** (mmHg)	112.6 ± 11.8	111.0 ± 11.6	112.1 ± 11.8	<0.001
**Diastolic BP** (mmHg)	71.0 ± 8.1	70.0 ± 8.0	70.7 ± 8.1	<0.001
**Unimpaired health**	99250 (75.6)	40670 (69.6)	139920 (73.8)	<0.001

**Israeli born**	110928 (84.6)	45896 (78.5)	156824 (82.7)	<0.001
Adolescent BMI (kg/m^2)				
	21.3 ± 3.1	21.7 ± 3.5	21.4 ± 3.2	<0.001
Underweight	61170 (4.7)	2761 (4.7)	8878 (4.7)	
Normal weight	112675 (85.9)	48118 (82.3)	160793 (84.8)	<0.001
Overweight	10079 (7.7)	5708 (9.8)	15787 (8.3)	
Obese	2336 (1.8)	1869 (3.2)	4205 (2.2)	
Pre-pregnancy BMI (kg/m2)				
Underweight	N/A	4547 (7.8)	4547 (2.4)	
Normal weight	N/A	38305 (65.5)	38305 (20.2)	
Overweight	N/A	10528 (18.0)	10528 (5.6)	-
Obese	N/A	5076 (8.7)	5076 (2.7)	
Age at first pregnancy**, **years				
Mean age	31.0 ± 4.6	31.0 ± 5.0	31.0 ± 4.7	<0.001
18 – 24	10110 (7.7)	5352 (9.2)	15462 (8.2)	
25 – 29	49069 (37.4)	22588 (38.6)	71657 (37.8)	
30 – 34	46985 (35.8)	18982 (32.5)	65967 (34.8)	<0.001
35 – 39	20289 (15.5)	8228 (14.1)	28517 (15.0)	
40 – 49	4754 (3.6)	3306 (5.7)	8060 (4.2)	
GIT Z-Score				
Low	14548 (11.1)	7431 (12.7%)	21979 (11.6)	
Intermediate	90758 (69.2)	39457 (67.5%)	130215 (68.7)	0.001
High	25901 (19.7)	11568 (19.8%)	37,469 (19.8)	
**GDM Diagnosis**	6900 (5.3)	3287 (5.6)	10187 (5.4)	<0.001

Dichotomous variables are reported as N (%), continuous variables are reported as mean ± standard deviation

GDM, gestational diabetes mellitus, BMI, body mass index; BP, blood pressure; BMI categories: underweight (BMI<5th percentile), normal (5th≤BMI<85th percentiles), overweight (85th≤BMI<95th percentiles), obese (≥95th percentile);
Abbreviations: BMI, body mass index; BP, blood pressure; GDM, gestational diabetes mellitus; GIT, General Intelligence Test; SD, standard deviation; cm, centimeters; mmHg, millimeters of mercury; N/A, not applicable.
