# Supplementary material for: Adolescent cognitive function and risk of gestational diabetes mellitus: A retrospective population-based cohort study
Source: PLoS One. 2026 Jul 17;21(7):e0351780. doi: 10.1371/journal.pone.0351780 (PMC13379011; doi:10.1371/journal.pone.0351780)
Supplement: Attachment — Submitted filename: Response - PLOS One - 2026-03.docx [file pone.0351780.s007.docx]

# Reviewer 1

This is an interesting study examining the association between adolescent cognitive function and the risk of gestational diabetes in a large cohort. The following comments are intended to improve the clarity and reporting of the manuscript.

**Response**: We thank the reviewer for the positive assessment of our work and for the constructive comments aimed at improving the clarity and reporting of the manuscript. We have carefully addressed all points raised, as detailed below.

The manuscript does not contain line numbers, which makes it difficult to reference specific sections during review. Please consider adding line numbers in the revised version to facilitate review and indicate where revisions have been made.

The manuscript would also benefit from careful proofreading to correct minor typographical and grammatical errors.

**Response**: The manuscript has been carefully proofread and revised throughout to correct typographical and grammatical errors and improve overall clarity and consistency.

Comments:

1. Title: Please clearly state this is a retrospective population- based cohort study

**Response**: We thank the reviewer for this important suggestion. We agree that the title should explicitly reflect the study design and have revised it accordingly to:

*Adolescent cognitive function and risk of gestational diabetes mellitus: a retrospective population-based cohort study*

2. Abstract:

Method: Please improve the sentence to describe it’s a retrospective population- based cohort study in the abstract and throughout the manuscript.

**Response**: We thank the reviewer for this helpful suggestion. The manuscript has been revised accordingly in both the abstract and the main text. The Methods section now reads:

**Methods**: Retrospective nationwide population-based cohort study linking the

Israel Defense Forces conscription database, in which cognitive data were collected at age ~17 years (1976–2016) with the electronic medical records of Maccabi Healthcare Services, containing prenatal and GDM screening data.

Please clearly describe the data sources used in the study, including the military cognitive assessment registry and the database used to identify pregnancies and GDM diagnoses.

**Response**: We thank the reviewer for this important comment. The data sources have been clarified in the Methods section of both the abstract and manuscript, explicitly detailing the Israel Defense Forces conscription database for adolescent cognitive assessment and the Maccabi Healthcare Services electronic medical records for identification of pregnancies and GDM outcomes (see revised Methods excerpt above).

3. Introduction:

-The introduction provides a concise overview of gestational diabetes and its potential long-term implications. However, it may benefit from a slightly expanded background to better contextualise the research question

**Response**: We appreciate the reviewer’s perspective on the Introduction. We have accordingly revised it to provide additional background on the public health relevance of GDM, the association between cognitive function and metabolic health, and the rationale for examining this relationship in the context of GDM, as detailed in the comments below.

-1st paragraph: Consider including information on the global, regional, or local prevalence of GDM to better highlight its public health burden.

**Response**:

Thank you. We revised the first paragraph to better emphasize the public health burden of GDM by including information on its global prevalence. The revised text now reads:

Gestational diabetes mellitus (GDM) is a significant health concern, affecting approximately 1 in 7 pregnancies globally, and is linked to numerous adverse maternal and neonatal outcomes, including macrosomia, birth trauma, and an increased risk of operative delivery

-2nd paragraph: Briefly summarise existing evidence linking cognitive function—particularly in adolescence—to metabolic disorders such as type 2 diabetes mellitus, including reported effect estimates where available.

**Response**: Thank you for this important comment. We revised the second paragraph to better summarize the existing literature linking cognitive function with adverse metabolic and cardiovascular outcomes. The revised text now states:

Differences in cognitive function have been linked with a range of adverse health outcomes, including all-cause mortality, metabolic impairment (i.e., type 2 diabetes mellitus) and cardiovascular disease.[7–10] Several mechanisms may underlie this association, including health-related behaviors, adherence to preventive care, socioeconomic trajectories, and shared developmental or biological determinants that may influence both cognitive and metabolic health.[11,12]

Regarding the inclusion of specific reported effect estimates, we respectfully felt that detailed quantitative comparisons would be more appropriate in the Discussion section, where our findings can be interpreted in the context of prior literature.

-Consider elaborating on the potential pathways through which adolescent cognitive function may be associated with the risk of GDM.

**Response**: Thank you. We agree that potential mechanisms for the examined relationship are important to consider. Accordingly, we expanded the Introduction to briefly mention several plausible pathways, including health-related behaviors, adherence to preventive care, socioeconomic trajectories, and shared developmental or biological determinants. We also chose to discuss these mechanisms in greater detail in the Discussion section, where they can be interpreted in the context of our findings. In this manner, we aimed to keep the Introduction concise while still providing sufficient rationale for the research question.

-The statement that “the association between cognitive function and GDM has yet to be established” is appropriate; however, may consider briefly expanding this point to better clarify the existing evidence gap and the rationale for the current study.

**Response**: Thank you for this suggestion. We revised the final part of the Introduction to more clearly define the evidence gap and strengthen the rationale for the present study. The revised text now reads:

Since GDM may represent an early clinical manifestation of underlying insulin resistance and metabolic vulnerability,[13] it is plausible that adolescent cognitive function may also be associated with the risk of GDM. However, this relationship has yet to be investigated. Examining the association between adolescent cognitive performance and later GDM may thereby provide further insight into the increasingly recognized interplay between cognition and metabolic health. Therefore, in this study, we aimed to evaluate the association between late adolescent cognitive score and subsequent GDM during the first pregnancy among a large population-based cohort.

4. Methods:

2.1 Study design and population: please state whether the study included all individual with a first documented pregnancy.

**Response**: We thank the reviewer for this helpful comment. We agree that the original wording was unclear. To improve clarity and precision, we revised the text using the reviewer’s suggested wording. We also further clarified later in the Methods that the primary outcome was GDM during the first documented pregnancy, and that for women with multiple pregnancies, only the first documented pregnancy in the MHS database was included in the analysis. The revised text now reads:

The study included all women in the MHS database who had undergone adolescent prerecruitment evaluation and later had a documented pregnancy with gestational diabetes screening using the glucose challenge test (GCT), as previously described.[15]

The primary outcome of this study was diagnosis of GDM during the first pregnancy documented in the MHS database.

The description of the study population is unclear. The sentence “This study included all individuals with a documented pregnancy in MHS, ascertained by the first glucose challenge test (GCT) who underwent adolescent prerecruitment evaluation” is difficult to interpret. Please clarify whether this was what you tried to explain. “The study included all women in the MHS database who had undergone adolescent prerecruitment evaluation and later had a documented first pregnancy with gestational diabetes screening using the glucose challenge test (GCT).

**Response**: We thank the reviewer for this comment. We agree that the original sentence was unclear and therefore adopted the reviewer’s suggested wording as detailed above.

2.2 Baseline demographic

-Education was dichotomized as ≤11 versus ≥12 years of schooling “as previously reported.” Please explain the rationale for this cut-off instead of stating “as previously reported”.

**Response**: We thank the reviewer for this important comment. To clarify the rationale for this categorization, we revised the text to specify that 12 years or more of formal schooling corresponds to completion of high school in Israel, whereas ≤11 years indicates non-completion. The revised text now reads:

Education was dichotomized into two categories ≤11 years or 12 years of formal schooling, with the latter corresponding to completion of high school, as previously reported.[10,15]

-Socioeconomic status was classified into three categories: low (1-3), intermediate (5-7) and high (8-10). Please describe the source of this socioeconomic scale, confirm whether the category ranges are correct- score 4 is missing.

**Response**: We thank the reviewer for identifying this error. Score 4 was inadvertently omitted from the text for low socioeconomic status category, and this has now been corrected. We also revised the text to better describe the source and basis of the socioeconomic scale. Specifically, residential socioeconomic status was based on the Israeli Bureau of Statistics classification of localities in Israel, which uses an ordinal scale from 1 to 10 reflecting area-level socioeconomic characteristics. This categorization has also been used in previous studies, including investigations of socioeconomic status in Israel in the context of cardiometabolic health, which delve into more detail on this categorization. The revised text now reads:

Residential socioeconomic status ranges on an ordinal scale of 1-10, reflecting the socioeconomic characteristics of each locality in Israel, as defined by the Israeli Bureau of Statistics. Socioeconomic status was classified into three categories: low (1-4), intermediate (5-7) and high (8-10), as detailed in previous investigations.[18]

2.3 Cognitive assessment

The categorisation of GIT Z-scores requires clarification. Please confirm whether the intended threshold for low-GIT is Z < −1.

**Response**: We thank the reviewer for identifying this point. The intended threshold for the low-GIT category was indeed Z < −1, and the text has been reviewed and revised accordingly.

2.4 GDM screening data

-The diagnostic thresholds for GDM should be clearly specified using explicit inequality signs (e.g., ≥ or >).

**Response**: The diagnostic thresholds are presented using explicit inequality signs in the revised manuscript, and we carefully reviewed this section again to ensure consistency and clarity throughout.

-Diagnostic criteria for GDM might have evolved over time, please clarify whether the same criteria were used throughout the study period and how potential temporal variation in diagnostic practice was handled.

**Response**: We thank the reviewer for this important comment. We agree that diagnostic practices may have varied over time. To address this, GDM was defined in our study based on t recorded glucose values from the two-step screening approach using prespecified quantitative thresholds, rather than relying solely on a documented clinical diagnosis. This approach allows for a more standardized outcome definition across the study period and reduced the impact of potential temporal variation in diagnostic practices.

2.5 Statistical analysis

-the manuscript reports odds ratios for the “prevalence” of GDM. Please revise the wording to either occurrence or incident of GDM.

**Response**: We thank the reviewer for this comment. We agree that “prevalence” was not the appropriate term in this context. The wording has been revised throughout the manuscript to refer to the occurrence or incidence of GDM, as appropriate.

-Although adjustment was done for several sociodemographic variables, adolescent cognitive performance is closely associated with lifelong socioeconomic conditions and health behaviors that may also influence the risk of GDM. Residual confounding by unmeasured factors such as lifestyle behaviors and adult socioeconomic status may therefore remain. Given the relatively modest effect sizes observed, this limitation should be acknowledged more explicitly in the discussion.

**Response**: We thank the reviewer for this important comment. We agree that, given the relatively modest effect sizes observed, the possibility of residual confounding by unmeasured socioeconomic and behavioral factors should be acknowledged more explicitly. We have therefore open the paragraph addressing potential explanations for the observed association with this matter. We did however revise the final portion of the text on this issue to reflect the potential dynamics in socioeconomic circumstances between adolescence and pregnancy. The revised text now reads:

The observed association between late adolescent cognitive function and incidence of GDM could have several explanations. First, this association may be principally mediated by the link between cognitive function and social determinants of health including socioeconomic status and maternal education. Low socioeconomic status has been previously linked with adverse pregnancy outcomes, including but not limited to GDM.[37,38] Although our results remained consistent when controlling for various covariates, including socioeconomic status and education, residual confounding by unmeasured socioeconomic factors may also play a role in mediating this relationship. **Moreover, because these variables were assessed in adolescence, they may not fully capture socioeconomic circumstances later in adulthood or at the time of pregnancy.**

5. Results

-The presentation of baseline characteristics in the text is difficult to follow due to the large number of percentages reported within a single sentence. Consider restructuring the sentence to first describe the overall pattern across GIT Z-score groups and then present the corresponding percentages for each group to improve readability.

Suggested version: Participants with higher GIT Z-scores were more likely to have ≥12 years of education (91.9%, 98.2%, and 99.5% in the low, intermediate, and high groups, respectively), high residential socioeconomic status……., and unimpaired health at adolescent……

**Response**: We thank the reviewer for this helpful suggestion. We therefore revised the text to first describe the overall pattern across GIT Z-score groups and then present the corresponding percentages, in line with the reviewer’s recommendation. The revised text now reads as follows:

Table 1 depicts the baseline characteristics of the study population, grouped by GIT Z-score. Mean age at first assessment was 17.3 ± 0.4 years. The proportion of women with obese adolescent BMI was 3.7% in the low GIT Z-score group, 2.2% in the intermediate group and 1.5% in the high group (p<0.001). Participants with higher GIT Z-scores had a lower proportion of obesity in adolescence and higher proportions of ≥12 years of formal education, high residential socioeconomic status, and unimpaired health. Specifically, the proportions of obese adolescent BMI were 3.7%, 2.2%, and 1.5% across the low, intermediate, and high GIT groups, respectively (p<0.001); the corresponding proportions for ≥12 years of formal education were 91.9%, 98.2%, and 99.5% (p<0.001), for high residential socioeconomic status 19.6%, 29.7%, and 38.0% (p<0.001), and for unimpaired health 69.7%, 74.1%, and 75.2% (p<0.001).

-Figure 2 clearly presents the odds ratios and confidence intervals for the regression models. For completeness, the authors may consider providing a table with the exact regression estimates and p-values to complement the figure.

**Response**: We thank the reviewer for this thoughtful suggestion. Respectfully, given the very large sample size of our cohort, we believe that the 95% confidence intervals presented in Figure 2 already adequately convey the precision of the estimates and their statistical significance. In all analyses shown, p-values were <0.001, which would be expected given the sample size and the magnitude of the observed estimates. We therefore do not believe that adding exact p-values would provide meaningful additional information, and it would likely add unnecessary clutter.

With regard to presenting the regression estimates for all covariates in an accompanying table, we chose not to do so because this may be misleading and could contribute to the well-described Table 2 fallacy. Namely, the inappropriate interpretation of covariate coefficients from a multivariable model as if they represent primary exposure effects, even though these estimates are typically included for adjustment rather than for etiologic interpretation. Since the purpose of these models was to estimate the association between adolescent cognitive score and GDM, we believe that focusing presentation on the primary exposure of interest improves clarity and avoids potential misinterpretation.

The x-axis label indicating “log scale” appears misleading, as the values shown correspond to odds ratios rather than log-transformed estimates. Please consider revising the axis label.

**Response**: We thank the reviewer for this comment. We respectfully note that the appropriateness of using a logarithmic vs. linear scale for forest plots of odds ratios has been specifically discussed in the paper cited below (Cruz-Retamozo et al.).

In that discussion, the authors explain that odds ratios are multiplicative measures centred around the null value of 1, and that a linear scale may produce a misleading visual impression because reciprocal values such as 0.5 and 2 are not displayed symmetrically around the null. They further note that this may distort the apparent magnitude and precision of associations, particularly for estimates below 1. Accordingly, they argue that when forest plots present measures of association such as odds ratios, risk ratios, or prevalence ratios, a logarithmic scale is the more appropriate choice. In line with this rationale, we consider the log scale methodologically appropriate for the present figure and have clarified the axis labelling to avoid confusion.

Cruz-Retamozo X, Prado-Ghezzi D, Pereyra-Elías R. Forest Plots: Linear or Logarithmic Scale? Journal of Adolescent Health. 2017;61:664–665.

**https://www.jahonline.org/article/S1054-139X(17)30424-X/fulltext**

6. Discussion

Previous studies cited by the authors reported larger effect estimates (approximately 1.4–1.7), whereas the associations observed in the current study are more modest. It would strengthen the discussion if the authors briefly addressed potential reasons for this difference, such as variations in population characteristics etc.

**Response**: We thank the reviewer for this thoughtful comment and agree that the more modest effect estimates observed in our study warrant further discussion. While the original paper mentioned the more modest estimated found in our study, we have now revised the Discussion to better address potential reasons for the difference from prior studies. The revised text now reads:

Although type 2 diabetes mellitus and GDM share several underlying pathophysiological mechanisms,[2,33] GDM remains a distinct entity which may often present as the first clinical manifestation of dysglycemia. To the best of our knowledge this is the first study evaluating the association between late adolescent cognitive function and the risk for subsequent GDM. While the associations observed in our study were weaker than those reported in studies examining type 2 diabetes mellitus, this may be explained by the more heterogeneous and transient nature of GDM, which is shaped not only by underlying metabolic vulnerability but also by pregnancy-specific physiological changes and obstetric factors.[34,35] In addition, GDM occurs earlier in the natural history of dysglycemia and may therefore reflect a less pronounced metabolic phenotype than overt type 2 diabetes. Nevertheless, the consistency of our findings across multiple adjusted models and sensitivity analyses suggests that, at the population level, lower cognitive function may indeed be associated with risk of GDM. Given the growing body of evidence linking glucose intolerance in pregnancy with future maternal[1,4,5] and child[36–38] cardiometabolic health, the findings of the current study provide additional support to the concept that cognitive function during adolescence could serve as an early indicator for metabolic risk.

Limitations: Cognitive performance was assessed once during adolescent prerecruitment evaluation, whereas the outcome occurred several years later during pregnancy. Cognitive ability and related socioeconomic circumstances may change over time, and a single measurement at adolescence may not fully capture cognitive function at the time of pregnancy. This potential exposure misclassification could attenuate the observed association and may be worth acknowledging as an additional limitation.

**Response**: We thank the reviewer for this important comment. We agree that the time interval between adolescent cognitive assessment and pregnancy should be acknowledged more explicitly and have added the following sentence earlier in the discussion, as previously mentioned:

Moreover, because these variables were assessed in adolescence, they may not fully capture socioeconomic circumstances later in adulthood or at the time of pregnancy.

# Reviewer 2

Remarks to the Author:

Tomer Talmy et al. report findings from a nationwide population-based cohort study examining the association between adolescent cognitive function and the risk of gestational diabetes mellitus (GDM) in the first pregnancy. Among 189,663 women, lower cognitive scores were associated with GDM risk compared with high scores (OR 1.17 for low and 1.09 for intermediate). These findings suggest that lower adolescent cognitive function may be an early marker of increased GDM risk. Given the limited findings of previous studies, this research carries important implications. Nevertheless, some descriptions in the manuscript are confusing and need to be revised, as detailed below.

**Response**: We thank the reviewer for the thoughtful and constructive comments. We are grateful for the positive assessment of our study and for recognizing the potential importance of these findings. We have carefully reviewed all of the points raised and did our best to address each comment thoroughly in the revised manuscript.

Comments:

1. Table 1 should include the gestational weeks at which GDM was diagnosed.

**Response**: We thank the reviewer for this helpful suggestion. We have now added gestational age at first GDM screening to Table 1. Although data for this variable were missing for approximately 20% of participants, the median values were very similar across groups. While the difference was statistically significant, likely owing to the large sample size, we do not consider it clinically meaningful.

Gestational age at first GDM screening in weeks was as follows: 25.0 (24.1–26.1), 24.9 (24.0–26.0), and 24.9 (24.0–25.9) in the low, intermediate, and high GIT Z-score groups, respectively; overall 24.9 (24.0–26.0).

We also added a corresponding footnote to clarify the extent of missing data for this variable:

Data regarding the timing of the first GDM examination were missing for 4,389 (20.0%) participants in the low Z-score group, 23,026 (17.7%) in the intermediate group, and 7,142 (19.1%) in the high Z-score group.

2. It is unclear how missing data, for example socioeconomic status, were handled. The authors should clarify their approach in the method section.

**Response**: We thank the reviewer for this important comment. We agree that the handling of missing data should be stated more clearly. We have therefore clarified in the Methods section that missing data were not imputed and that a complete-case approach was used for each model. The revised text now reads:

Missing data were not imputed, and therefore, observations with missing data for any variable included in a given model were excluded from the aforementioned analysis. This

In addition, we clarified the extent of missing data in the figure caption for the fully adjusted model (Model 3):

Overall, 1,080 observations (0.6%) had missing data for at least one covariate and were therefore excluded from the final model.

3. Given that GIT is divided into low, intermediate, and high categories, a trend test (P-trend) should be also included to assess potential dose-response relationships.

**Response**: We thank the reviewer for this helpful suggestion. In accordance with this comment, we added a test for trend to assess the dose-response relationship across GIT categories. In the Methods, we now state:

Finally, a linear regression model was fitted to assess the p-trend, with GIT Z-score treated as a continuous variable.

We also added the corresponding result to the Results section:

Overall, 10,187 (5.4%) individuals were diagnosed with GDM, with a rate of 5.5% (n=1,209), 5.4% (n=7,079) and 5.0% (n=1,889) for women in the low, intermediate and high GIT Z-score groups, respectively (p-trend<0.001).

4. The role of family history of diabetes in the observed associations should be discussed or accounted for in the analysis.

**Response**: We did not have access to family history to account for it, and have mentioned this in the limitations –

Sixth, several potentially relevant determinants of GDM risk, including family history of cardiometabolic disease, were unavailable for adjustment. In addition, although we adjusted for multiple socioeconomic covariates, these were measured in adolescence and therefore may not fully capture socioeconomic conditions later in adulthood or at the time of pregnancy.

5. In Table S1, the authors compare women with and without pre-pregnancy BMI data. It would be more informative to compare the subcohort with BMI data to the overall population and report any differences in GIT Z-scores and GDM incidence.

**Response**: We appreciate the reviewer’s suggestion. In accordance with this comment, we added an overall cohort column to Supplementary Table 1, allowing direct comparison of women with available pre-pregnancy BMI data, women without such data, and the overall study population. We have also included the GIT Z-score and GDM variables in the revised table.

6. Figure S3 is described as a subgroup analysis, but it is more appropriately considered a sensitivity analysis. Further stratified analyses by BMI status (e.g., overweight vs. non-overweight) should be considered.

**Response**: We thank the reviewer for this helpful comment. We agree that the analysis presented in Supplementary Figure 3 is more appropriately considered a sensitivity analysis, and we have revised the terminology throughout the manuscript accordingly. In addition, as suggested, we performed an additional stratified analysis by adolescent BMI category.

The corresponding result was added to the Results section as follows:

In analyses stratified by adolescent BMI groups (Supplementary Figure 4), estimates of the association between lower GIT Z-score and GDM were most pronounced among women with overweight and obesity, whereas weaker and non-significant associations were observed in the underweight group.

7. Since GIT includes four subdomains, the authors should examine the associations between each subdomain and GDM separately to determine whether different cognitive domains have different effects.

**Response**: Although the overall cognitive score has been systematically recorded for decades, data on specific cognitive subdomains are incomplete and contain substantial missingness. While subdomain-level assessments have been analyzed in selected prior studies, these data were not available for the current cohort, particularly within the cross-linked dataset with Maccabi that includes GDM outcomes. Consequently, we were unable to perform subdomain-specific analyses. We agree that such data could provide additional insight and have therefore acknowledged this limitation in the manuscript.

Relevant excerpt -

Fifth, although an association was observed between overall cognitive score and GDM, data on specific cognitive subdomains were unavailable, precluding analysis of individual GIT components, which may have distinct relationships with metabolic risk.

8. Although the associations between GIT and GDM were statistically significant, the adolescent cognitive assessment was conducted long before GDM diagnosis. Lifestyle factors may have a stronger influence on GDM, and the conclusions should therefore be interpreted with caution.

**Response**: We thank the reviewer for this important comment and agree that the findings should be interpreted with appropriate caution, given the interval between adolescent cognitive assessment and pregnancy, and the possible affects of lifestyle and other later-life factors on GDM risk. We have addressed this point more explicitly in the Limitations section, as follows:

Sixth, several potentially relevant determinants of GDM risk, including family history of cardiometabolic disease, were unavailable for adjustment. In addition, although we adjusted for multiple socioeconomic covariates, these were measured in adolescence and therefore may not fully capture socioeconomic conditions later in adulthood or at the time of pregnancy.
