# Supplementary material for: Adolescent cognitive function and risk of gestational diabetes mellitus: A retrospective population-based cohort study
Source: PLoS One. 2026 Jul 17;21(7):e0351780. doi: 10.1371/journal.pone.0351780 (PMC13379011; doi:10.1371/journal.pone.0351780)
Supplement: Attachment — Submitted filename: R2 - Response - PLOS One - 2026-05.docx [file pone.0351780.s008.docx]

# Associate Editor

The authors have substantially addressed the reviewers’ required revisions, and the manuscript is much clearer and better contextualized as a result. The study design, data sources, handling of missing data, additional trend and stratified analyses, and strengthened discussion of residual confounding and unavailable covariates are all adequately addressed. I noted only minor residual issues: the limitation related to the long interval between adolescent cognitive assessment and later GDM could be stated more directly in the manuscript itself, and the description of the p-trend analysis may need slight clarification. Overall, these are small points, and in my view the revision is responsive and suitable to proceed without another major round.

**Response**: We thank the Associate Editor for the careful evaluation of our revised manuscript and for the positive assessment of the revision. We are grateful for the recognition that the major points raised by the reviewers were adequately addressed.

In response to the remaining minor comments, we made two additional clarifications in the manuscript. First, we revised the Limitations section to state more directly that adolescent cognitive function was assessed many years before pregnancy, and that a single measurement in adolescence may not fully reflect cognitive function at the time of GDM occurrence. Second, we clarified the description of the p-trend analysis in the Methods section to improve precision and readability.

Specifically, we added the following text to the limitations section:

Likewise, cognitive function was assessed once during adolescence, several years before pregnancy, and this single measurement may not fully reflect cognitive function at the time of pregnancy.

And revised the Methods as follows –

Finally, a linear regression model was fitted to assess the linear relationship between GIT Z-score and GDM incidence, with GIT Z-score treated as a continuous variable.

# Reviewer 1

Reviewer #1: The authors have adequately addressed the reviewer comments. One minor point remains: the study design sentence in the abstract (Lines 53–56) could be revised for clarity and readability, as the current wording is somewhat difficult to follow.

**Response**: We thank the reviewer for this helpful comment. We agree that the original study design sentence in the abstract could be improved for clarity and readability. We therefore revised the Methods section of the abstract as follows:

In this retrospective nationwide population-based cohort study, data from the Israel Defense Forces conscription database, including cognitive assessments conducted at approximately age 17 years (1976–2016), were linked with electronic medical records from Maccabi Healthcare Services, documenting prenatal care and gestational diabetes screening data.
