# Supplementary material for: Adolescent cognitive function and risk of gestational diabetes mellitus: A retrospective population-based cohort study
Source: PLoS One. 2026 Jul 17;21(7):e0351780. doi: 10.1371/journal.pone.0351780 (PMC13379011; doi:10.1371/journal.pone.0351780)
Supplement: Peer review history

# Peer review: Decision Letter 0

**DOI:** 10.1371/journal.pone.0351780.r001

**Authors:** Melvin Marzan

22 Mar 2026

PONE-D-25-60369

Adolescent Cognitive Function and Risk of Gestational Diabetes Mellitus: A Population-Based Cohort Study

PLOS One

Dear Dr. Talmy,

Thank you for submitting your manuscript to PLOS ONE. After careful consideration, we feel that it has merit but does not fully meet PLOS ONE’s publication criteria as it currently stands. Therefore, we invite you to submit a revised version of the manuscript that addresses the points raised during the review process.

==============================

Please ensure that all comments raised by both reviewers are carefully and thoroughly addressed in your revised manuscript and point-by-point response letter

==============================

Please submit your revised manuscript by May 06 2026 11:59PM. If you will need more time than this to complete your revisions, please reply to this message or contact the journal office at plosone@plos.org. When you're ready to submit your revision, log on to https://www.editorialmanager.com/pone/ and select the 'Submissions Needing Revision' folder to locate your manuscript file.

Please include the following items when submitting your revised manuscript:

- A letter that responds to each point raised by the academic editor and reviewer(s). You should upload this letter as a separate file labeled 'Response to Reviewers'.
- A marked-up copy of your manuscript that highlights changes made to the original version. You should upload this as a separate file labeled 'Revised Manuscript with Track Changes'.
- An unmarked version of your revised paper without tracked changes. You should upload this as a separate file labeled 'Manuscript'.

If you would like to make changes to your financial disclosure, please include your updated statement in your cover letter. Guidelines for resubmitting your figure files are available below the reviewer comments at the end of this letter.

If applicable, we recommend that you deposit your laboratory protocols in protocols.io to enhance the reproducibility of your results. Protocols.io assigns your protocol its own identifier (DOI) so that it can be cited independently in the future. For instructions see: https://journals.plos.org/plosone/s/submission-guidelines#loc-laboratory-protocols. Additionally, PLOS ONE offers an option for publishing peer-reviewed Lab Protocol articles, which describe protocols hosted on protocols.io. Read more information on sharing protocols at https://plos.org/protocols?utm_medium=editorial-email&utm_source=authorletters&utm_campaign=protocols.

We look forward to receiving your revised manuscript.

Kind regards,

Melvin Marzan, BSc, MSc TM, PhD

Academic Editor

PLOS One

Journal Requirements:

When submitting your revision, we need you to address these additional requirements.

1. Please ensure that your manuscript meets PLOS ONE's style requirements, including those for file naming. The PLOS ONE style templates can be found at

https://journals.plos.org/plosone/s/file?id=wjVg/PLOSOne_formatting_sample_main_body.pdf and

https://journals.plos.org/plosone/s/file?id=ba62/PLOSOne_formatting_sample_title_authors_affiliations.pdf

2. Please update your submission to use the PLOS LaTeX template. The template and more information on our requirements for LaTeX submissions can be found at http://journals.plos.org/plosone/s/latex.

3. Thank you for stating the following in the Acknowledgments Section of your manuscript:

“This research was funded by internal departmental sources and received no external funding.”

We note that you have provided funding information that is not currently declared in your Funding Statement. However, funding information should not appear in the Acknowledgments section or other areas of your manuscript. We will only publish funding information present in the Funding Statement section of the online submission form.

Please remove any funding-related text from the manuscript and let us know how you would like to update your Funding Statement. Currently, your Funding Statement reads as follows:

“The author(s) received no specific funding for this work.”

Please include your amended statements within your cover letter; we will change the online submission form on your behalf.

4. In the online submission form you indicate that your data is not available for proprietary reasons and have provided a contact point for accessing this data. Please note that your current contact point is a co-author on this manuscript. According to our Data Policy, the contact point must not be an author on the manuscript and must be an institutional contact, ideally not an individual. Please revise your data statement to a non-author institutional point of contact, such as a data access or ethics committee, and send this to us via return email. Please also include contact information for the third party organization, and please include the full citation of where the data can be found.

5. Your ethics statement should only appear in the Methods section of your manuscript. If your ethics statement is written in any section besides the Methods, please move it to the Methods section and delete it from any other section. Please ensure that your ethics statement is included in your manuscript, as the ethics statement entered into the online submission form will not be published alongside your manuscript.

6. Please include a separate caption for each figure in your manuscript.

7. If the reviewer comments include a recommendation to cite specific previously published works, please review and evaluate these publications to determine whether they are relevant and should be cited. There is no requirement to cite these works unless the editor has indicated otherwise.

[Note: HTML markup is below. Please do not edit.]

Reviewers' comments:

Reviewer's Responses to Questions

**Comments to the Author**

1. Is the manuscript technically sound, and do the data support the conclusions?

The manuscript must describe a technically sound piece of scientific research with data that supports the conclusions. Experiments must have been conducted rigorously, with appropriate controls, replication, and sample sizes. The conclusions must be drawn appropriately based on the data presented.

Reviewer #1: Yes

Reviewer #2: Yes

**********

2. Has the statistical analysis been performed appropriately and rigorously?

Reviewer #1: Yes

Reviewer #2: Yes

**********

3. Have the authors made all data underlying the findings in their manuscript fully available?

The PLOS Data policy requires authors to make all data underlying the findings described in their manuscript fully available without restriction, with rare exception (please refer to the Data Availability Statement in the manuscript PDF file). The data should be provided as part of the manuscript or its supporting information, or deposited to a public repository. For example, in addition to summary statistics, the data points behind means, medians and variance measures should be available. If there are restrictions on publicly sharing data—e.g. participant privacy or use of data from a third party—those must be specified.

Reviewer #1: Yes

Reviewer #2: No

**********

4. Is the manuscript presented in an intelligible fashion and written in standard English?

PLOS ONE does not copyedit accepted manuscripts, so the language in submitted articles must be clear, correct, and unambiguous. Any typographical or grammatical errors should be corrected at revision, so please note any specific errors here.

Reviewer #1: Yes

Reviewer #2: Yes

**********

5. Review Comments to the Author

Please use the space provided to explain your answers to the questions above. You may also include additional comments for the author, including concerns about dual publication, research ethics, or publication ethics. (Please upload your review as an attachment if it exceeds 20,000 characters)

Reviewer #1: This is an interesting study examining the association between adolescent cognitive function and the risk of gestational diabetes in a large cohort. The following comments are intended to improve the clarity and reporting of the manuscript.

The manuscript does not contain line numbers, which makes it difficult to reference specific sections during review. Please consider adding line numbers in the revised version to facilitate review and indicate where revisions have been made.

The manuscript would also benefit from careful proofreading to correct minor typographical and grammatical errors.

Comments:

1. Title: Please clearly state this is a retrospective population- based cohort study

2. Abstract:

Method: Please improve the sentence to describe it’s a retrospective population- based cohort study in the abstract and throughout the manuscript.

Please clearly describe the data sources used in the study, including the military cognitive assessment registry and the database used to identify pregnancies and GDM diagnoses.

3. Introduction:

-The introduction provides a concise overview of gestational diabetes and its potential long-term implications. However, it may benefit from a slightly expanded background to better contextualise the research question.

-1st paragraph: Consider including information on the global, regional, or local prevalence of GDM to better highlight its public health burden.

-2nd paragraph: Briefly summarise existing evidence linking cognitive function—particularly in adolescence—to metabolic disorders such as type 2 diabetes mellitus, including reported effect estimates where available.

-Consider elaborating on the potential pathways through which adolescent cognitive function may be associated with the risk of GDM.

-The statement that “the association between cognitive function and GDM has yet to be established” is appropriate; however, may consider briefly expanding this point to better clarify the existing evidence gap and the rationale for the current study.

4. Methods:

2.1 Study design and population: please state whether the study included all individual with a first documented pregnancy.

The description of the study population is unclear. The sentence “This study included all individuals with a documented pregnancy in MHS, ascertained by the first glucose challenge test (GCT) who underwent adolescent prerecruitment evaluation” is difficult to interpret. Please clarify whether this was what you tried to explain. “The study included all women in the MHS database who had undergone adolescent prerecruitment evaluation and later had a documented first pregnancy with gestational diabetes screening using the glucose challenge test (GCT).

2.2 Baseline demographic

-Education was dichotomized as ≤11 versus ≥12 years of schooling “as previously reported.” Please explain the rationale for this cut-off instead of stating “as previously reported”.

-Socioeconomic status was classified into three categories: low (1-3), intermediate (5-7) and high (8-10). Please describe the source of this socioeconomic scale, confirm whether the category ranges are correct- score 4 is missing.

2.3 Cognitive assessment

The categorisation of GIT Z-scores requires clarification. Please confirm whether the intended threshold for low-GIT is Z < −1.

2.4 GDM screening data

-The diagnostic thresholds for GDM should be clearly specified using explicit inequality signs (e.g., ≥ or >).

-Diagnostic criteria for GDM might have evolved over time, please clarify whether the same criteria were used throughout the study period and how potential temporal variation in diagnostic practice was handled.

2.5 Statistical analysis

-the manuscript reports odds ratios for the “prevalence” of GDM. Please revise the wording to either occurrence or incident of GDM.

-Although adjustment was done for several sociodemographic variables, adolescent cognitive performance is closely associated with lifelong socioeconomic conditions and health behaviors that may also influence the risk of GDM. Residual confounding by unmeasured factors such as lifestyle behaviors and adult socioeconomic status may therefore remain. Given the relatively modest effect sizes observed, this limitation should be acknowledged more explicitly in the discussion.

5. Results

-The presentation of baseline characteristics in the text is difficult to follow due to the large number of percentages reported within a single sentence. Consider restructuring the sentence to first describe the overall pattern across GIT Z-score groups and then present the corresponding percentages for each group to improve readability.

Suggested version: Participants with higher GIT Z-scores were more likely to have ≥12 years of education (91.9%, 98.2%, and 99.5% in the low, intermediate, and high groups, respectively), high residential socioeconomic status……., and unimpaired health at adolescent……

-Figure 2 clearly presents the odds ratios and confidence intervals for the regression models. For completeness, the authors may consider providing a table with the exact regression estimates and p-values to complement the figure.

The x-axis label indicating “log scale” appears misleading, as the values shown correspond to odds ratios rather than log-transformed estimates. Please consider revising the axis label.

6. Discussion

Previous studies cited by the authors reported larger effect estimates (approximately 1.4–1.7), whereas the associations observed in the current study are more modest. It would strengthen the discussion if the authors briefly addressed potential reasons for this difference, such as variations in population characteristics etc.

Limitations: Cognitive performance was assessed once during adolescent prerecruitment evaluation, whereas the outcome occurred several years later during pregnancy. Cognitive ability and related socioeconomic circumstances may change over time, and a single measurement at adolescence may not fully capture cognitive function at the time of pregnancy. This potential exposure misclassification could attenuate the observed association and may be worth acknowledging as an additional limitation.

Reviewer #2: Review:

Remarks to the Author:

Tomer Talmy et al. report findings from a nationwide population-based cohort study examining the association between adolescent cognitive function and the risk of gestational diabetes mellitus (GDM) in the first pregnancy. Among 189,663 women, lower cognitive scores were associated with GDM risk compared with high scores (OR 1.17 for low and 1.09 for intermediate). These findings suggest that lower adolescent cognitive function may be an early marker of increased GDM risk. Given the limited findings of previous studies, this research carries important implications. Nevertheless, some descriptions in the manuscript are confusing and need to be revised, as detailed below.

Comments:

1. Table 1 should include the gestational weeks at which GDM was diagnosed.

2. It is unclear how missing data, for example socioeconomic status, were handled. The authors should clarify their approach in the method section.

3. Given that GIT is divided into low, intermediate, and high categories, a trend test (P-trend) should be also included to assess potential dose-response relationships.

4. The role of family history of diabetes in the observed associations should be discussed or accounted for in the analysis.

5. In Table S1, the authors compare women with and without pre-pregnancy BMI data. It would be more informative to compare the subcohort with BMI data to the overall population and report any differences in GIT Z-scores and GDM incidence.

6. Figure S3 is described as a subgroup analysis, but it is more appropriately considered a sensitivity analysis. Further stratified analyses by BMI status (e.g., overweight vs. non-overweight) should be considered.

7. Since GIT includes four subdomains, the authors should examine the associations between each subdomain and GDM separately to determine whether different cognitive domains have different effects.

8. Although the associations between GIT and GDM were statistically significant, the adolescent cognitive assessment was conducted long before GDM diagnosis. Lifestyle factors may have a stronger influence on GDM, and the conclusions should therefore be interpreted with caution.

**********

6. PLOS authors have the option to publish the peer review history of their article (what does this mean?). If published, this will include your full peer review and any attached files.

If you choose “no”, your identity will remain anonymous but your review may still be made public.

**Do you want your identity to be public for this peer review?**  For information about this choice, including consent withdrawal, please see our Privacy Policy.

Reviewer #1: No

Reviewer #2: No

**********

[NOTE: If reviewer comments were submitted as an attachment file, they will be attached to this email and accessible via the submission site. Please log into your account, locate the manuscript record, and check for the action link "View Attachments". If this link does not appear, there are no attachment files.]

To ensure your figures meet our technical requirements, please review our figure guidelines: https://journals.plos.org/plosone/s/figures

You may also use PLOS’s free figure tool, NAAS, to help you prepare publication quality figures: https://journals.plos.org/plosone/s/figures#loc-tools-for-figure-preparation.

NAAS will assess whether your figures meet our technical requirements by comparing each figure against our figure specifications.

# Author comment: Author response to Decision Letter 1

**DOI:** 10.1371/journal.pone.0351780.r002

21 Apr 2026

Response is attached.

Attachment Submitted filename: Response - PLOS One - 2026-03.docx

# Peer review: Decision Letter 1

**DOI:** 10.1371/journal.pone.0351780.r003

**Authors:** Melvin Marzan, Melvin Marzan

17 May 2026

PONE-D-25-60369R1

Adolescent Cognitive Function and Risk of Gestational Diabetes Mellitus: A Retrospective Population-Based Cohort Study

PLOS One

Dear Dr. Talmy,

Thank you for submitting your manuscript to PLOS ONE. After careful consideration, we feel that it has merit but does not fully meet PLOS ONE’s publication criteria as it currently stands. Therefore, we invite you to submit a revised version of the manuscript that addresses the points raised during the review process.

Please submit your revised manuscript by Jul 01 2026 11:59PM. If you will need more time than this to complete your revisions, please reply to this message or contact the journal office at plosone@plos.org. When you're ready to submit your revision, log on to https://www.editorialmanager.com/pone/ and select the 'Submissions Needing Revision' folder to locate your manuscript file.

Please include the following items when submitting your revised manuscript:

- A letter that responds to each point raised by the academic editor and reviewer(s). You should upload this letter as a separate file labeled 'Response to Reviewers'.
- A marked-up copy of your manuscript that highlights changes made to the original version. You should upload this as a separate file labeled 'Revised Manuscript with Track Changes'.
- An unmarked version of your revised paper without tracked changes. You should upload this as a separate file labeled 'Manuscript'.

If you would like to make changes to your financial disclosure, please include your updated statement in your cover letter. Guidelines for resubmitting your figure files are available below the reviewer comments at the end of this letter.

If applicable, we recommend that you deposit your laboratory protocols in protocols.io to enhance the reproducibility of your results. Protocols.io assigns your protocol its own identifier (DOI) so that it can be cited independently in the future. For instructions see: https://journals.plos.org/plosone/s/submission-guidelines#loc-laboratory-protocols. Additionally, PLOS ONE offers an option for publishing peer-reviewed Lab Protocol articles, which describe protocols hosted on protocols.io. Read more information on sharing protocols at https://plos.org/protocols?utm_medium=editorial-email&utm_source=authorletters&utm_campaign=protocols.

As the corresponding author, your ORCID iD is verified in the submission system and will appear in the published article. PLOS supports the use of ORCID, and we encourage all coauthors to register for an ORCID iD and use it as well. Please encourage your coauthors to verify their ORCID iD within the submission system before final acceptance, as unverified ORCID iDs will not appear in the published article. *Only*  the individual author can complete the verification step; PLOS staff *cannot*  verify ORCID iDs on behalf of authors.

We look forward to receiving your revised manuscript.

Kind regards,

Melvin Marzan, BSc, MSc TM, PhD

Academic Editor

PLOS One

Journal Requirements:

If the reviewer comments include a recommendation to cite specific previously published works, please review and evaluate these publications to determine whether they are relevant and should be cited. There is no requirement to cite these works unless the editor has indicated otherwise.

Please review your reference list to ensure that it is complete and correct. If you have cited papers that have been retracted, please include the rationale for doing so in the manuscript text, or remove these references and replace them with relevant current references. Any changes to the reference list should be mentioned in the rebuttal letter that accompanies your revised manuscript. If you need to cite a retracted article, indicate the article’s retracted status in the References list and also include a citation and full reference for the retraction notice.

Additional Editor Comments:

The authors have substantially addressed the reviewers’ required revisions, and the manuscript is much clearer and better contextualized as a result. The study design, data sources, handling of missing data, additional trend and stratified analyses, and strengthened discussion of residual confounding and unavailable covariates are all adequately addressed. I noted only minor residual issues: the limitation related to the long interval between adolescent cognitive assessment and later GDM could be stated more directly in the manuscript itself, and the description of the p-trend analysis may need slight clarification. Overall, these are small points, and in my view the revision is responsive and suitable to proceed without another major round.

[Note: HTML markup is below. Please do not edit.]

Reviewers' comments:

Reviewer's Responses to Questions

**Comments to the Author**

1. If the authors have adequately addressed your comments raised in a previous round of review and you feel that this manuscript is now acceptable for publication, you may indicate that here to bypass the “Comments to the Author” section, enter your conflict of interest statement in the “Confidential to Editor” section, and submit your "Accept" recommendation.

Reviewer #1: All comments have been addressed

Reviewer #2: All comments have been addressed

**********

2. Is the manuscript technically sound, and do the data support the conclusions?

The manuscript must describe a technically sound piece of scientific research with data that supports the conclusions. Experiments must have been conducted rigorously, with appropriate controls, replication, and sample sizes. The conclusions must be drawn appropriately based on the data presented.

Reviewer #1: Yes

Reviewer #2: Yes

**********

3. Has the statistical analysis been performed appropriately and rigorously?

Reviewer #1: Yes

Reviewer #2: Yes

**********

4. Have the authors made all data underlying the findings in their manuscript fully available?

The PLOS Data policy requires authors to make all data underlying the findings described in their manuscript fully available without restriction, with rare exception (please refer to the Data Availability Statement in the manuscript PDF file). The data should be provided as part of the manuscript or its supporting information, or deposited to a public repository. For example, in addition to summary statistics, the data points behind means, medians and variance measures should be available. If there are restrictions on publicly sharing data—e.g. participant privacy or use of data from a third party—those must be specified.

Reviewer #1: No

Reviewer #2: (No Response)

**********

5. Is the manuscript presented in an intelligible fashion and written in standard English?

PLOS ONE does not copyedit accepted manuscripts, so the language in submitted articles must be clear, correct, and unambiguous. Any typographical or grammatical errors should be corrected at revision, so please note any specific errors here.

Reviewer #1: Yes

Reviewer #2: Yes

**********

6. Review Comments to the Author

Please use the space provided to explain your answers to the questions above. You may also include additional comments for the author, including concerns about dual publication, research ethics, or publication ethics. (Please upload your review as an attachment if it exceeds 20,000 characters)

Reviewer #1: The authors have adequately addressed the reviewer comments. One minor point remains: the study design sentence in the abstract (Lines 53–56) could be revised for clarity and readability, as the current wording is somewhat difficult to follow.

Reviewer #2: (No Response)

**********

7. PLOS authors have the option to publish the peer review history of their article (what does this mean?). If published, this will include your full peer review and any attached files.

If you choose “no”, your identity will remain anonymous but your review may still be made public.

**Do you want your identity to be public for this peer review?**  For information about this choice, including consent withdrawal, please see our Privacy Policy.

Reviewer #1: No

Reviewer #2: No

**********

[NOTE: If reviewer comments were submitted as an attachment file, they will be attached to this email and accessible via the submission site. Please log into your account, locate the manuscript record, and check for the action link "View Attachments". If this link does not appear, there are no attachment files.]

To ensure your figures meet our technical requirements, please review our figure guidelines: https://journals.plos.org/plosone/s/figures

You may also use PLOS’s free figure tool, NAAS, to help you prepare publication quality figures: https://journals.plos.org/plosone/s/figures#loc-tools-for-figure-preparation.

NAAS will assess whether your figures meet our technical requirements by comparing each figure against our figure specifications.

# Author comment: Author response to Decision Letter 2

**DOI:** 10.1371/journal.pone.0351780.r004

31 May 2026

Respone included in attached file.

Attachment Submitted filename: R2 - Response - PLOS One - 2026-05.docx

# Editor report: Decision Letter 2

**DOI:** 10.1371/journal.pone.0351780.r005

**Authors:** Melvin Marzan, Melvin Marzan, Melvin Marzan

1 Jun 2026

Adolescent Cognitive Function and Risk of Gestational Diabetes Mellitus: A Retrospective Population-Based Cohort Study

PONE-D-25-60369R2

Dear Dr. Talmy,

We’re pleased to inform you that your manuscript has been judged scientifically suitable for publication and will be formally accepted for publication once it meets all outstanding technical requirements.

Within one week, you’ll receive an e-mail detailing the required amendments. When these have been addressed, you’ll receive a formal acceptance letter and your manuscript will be scheduled for publication.

An invoice will be generated when your article is formally accepted. Please note, if your institution has a publishing partnership with PLOS and your article meets the relevant criteria, all or part of your publication costs will be covered. Please make sure your user information is up-to-date by logging into Editorial Manager at Editorial Manager® and clicking the ‘Update My Information' link at the top of the page. For questions related to billing, please contact billing support.

If your institution or institutions have a press office, please notify them about your upcoming paper to help maximize its impact. If they’ll be preparing press materials, please inform our press team as soon as possible -- no later than 48 hours after receiving the formal acceptance. Your manuscript will remain under strict press embargo until 2 pm Eastern Time on the date of publication. For more information, please contact onepress@plos.org.

Kind regards,

Melvin Marzan, BSc, MSc TM, PhD

Academic Editor

PLOS One

Additional Editor Comments (optional):

Reviewers' comments:

# Editor report: Acceptance letter

**DOI:** 10.1371/journal.pone.0351780.r006

**Authors:** Melvin Marzan, Melvin Marzan, Melvin Marzan

PONE-D-25-60369R2

PLOS One

Dear Dr. Talmy,

I'm pleased to inform you that your manuscript has been deemed suitable for publication in PLOS One. Congratulations! Your manuscript is now being handed over to our production team.

At this stage, our production department will prepare your paper for publication. This includes ensuring the following:

* All references, tables, and figures are properly cited

* All relevant supporting information is included in the manuscript submission,

* There are no issues that prevent the paper from being properly typeset

You will receive further instructions from the production team, including instructions on how to review your proof when it is ready. Please keep in mind that we are working through a large volume of accepted articles, so please give us a few days to review your paper and let you know the next and final steps.

Lastly, if your institution or institutions have a press office, please let them know about your upcoming paper now to help maximize its impact. If they'll be preparing press materials, please inform our press team within the next 48 hours. Your manuscript will remain under strict press embargo until 2 pm Eastern Time on the date of publication. For more information, please contact onepress@plos.org.

You will receive an invoice from PLOS for your publication fee after your manuscript has reached the completed accept phase. If you receive an email requesting payment before acceptance or for any other service, this may be a phishing scheme. Learn how to identify phishing emails and protect your accounts at https://explore.plos.org/phishing.

If we can help with anything else, please email us at customercare@plos.org.

Thank you for submitting your work to PLOS ONE and supporting open access.

Kind regards,

PLOS ONE Editorial Office Staff

on behalf of

Dr. Melvin Marzan

Academic Editor

PLOS One
